# Network Pharmacology Identifies the Mechanisms of Action of Tongxie Anchang Decoction in the Treatment of Irritable Bowel Syndrome with Diarrhea Predominant

**DOI:** 10.1155/2020/2723705

**Published:** 2020-11-17

**Authors:** Xiang Tan, Wenjing Pei, Chune Xie, Zhibin Wang, Tangyou Mao, Xingjie Zhao, Fushun Kou, Qiongqiong Lu, Zhongmei Sun, Xiaoxuan Xue, Junxiang Li

**Affiliations:** ^1^Graduate School of Beijing University of Chinese Medicine, Beijing 100029, China; ^2^Department of Gastroenterology, Dongfang Hospital, Beijing University of Chinese Medicine, Beijing 100078, China

## Abstract

**Aim:**

This study aims to uncover the pharmacological mechanism of Tongxie Anchang Decoction (TXACD), a new and effective traditional Chinese medicine (TCM) prescription, for treating irritable bowel syndrome with diarrhea predominant (IBS-D) using network pharmacology.

**Methods:**

The active compounds and putative targets of TXACD were retrieved from TCMSP database and published literature; related target genes of IBS-D were retrieved from GeneCards; PPI network of the common target hub gene was constructed by STRING. Furthermore, these hub genes were analyzed using gene ontology (GO) enrichment analysis and Kyoto Encyclopedia of Genes and Genomes (KEGG) enrichment analysis.

**Results:**

A total of 54 active compounds and 639 targets were identified through a database search. The compound-target network was constructed, and the key compounds were screened out according to the degree. By using the PPI and GO and KEGG enrichment analyses, the pharmacological mechanism network of TXACD in the treatment of IBS-D was constructed.

**Conclusions:**

This study revealed the possible mechanisms by which TXACD treatment alleviated IBS-D involvement in the modulation of multiple targets and multiple pathways, including the immune regulation, inflammatory response, and oxidative stress. These findings provide novel insights into the regulatory role of TXACD in the prevention and treatment of IBS-D and hold promise for herb-based complementary and alternative therapy.

## 1. Introduction

Irritable bowel syndrome (IBS) is a very common functional gastrointestinal disease in clinic, which is characterized by abdominal pain accompanied by changes in defecation habits, but lack of gastrointestinal structural and biochemical abnormalities [1]. Irritable bowel syndrome affects 10%–20% of the population [2], which has a great impact on the life and work of patients. However, the pathogenesis of IBS is not clear. At present, it is generally believed that abnormal regulation of brain-gut axis, visceral hypersensitivity, imbalance of intestinal flora, gastrointestinal motility disorder, intestinal infection and inflammation, and mental and psychological factors are involved in the pathogenesis of IBS. Irritable bowel syndrome with diarrhea predominant (IBS-D) is the most common subtype of irritable bowel syndrome. Due to the heterogeneity of pathogenesis, the treatment of IBS-D is also diverse, including antidiarrheal treatment, gastrointestinal spasmolysis, probiotic supplement, antibiotics, and antidepressants. However, the effect of treatment is unstable and easy to relapse. Therefore, more effective and safe methods for the treatment of IBS-D are needed.

Traditional Chinese medicine (TCM) is a medical system with a long history and unique theories and technical methods. It has been widely used to treat a series of diseases for decades [3], with the advantages of low price and easy availability everywhere, and because many drugs are extracted from natural sources such as herbs, the side effects are less. Tongxie Anchang Decoction (TXACD) is composed of seven Chinese herbs, including Baizhu (Atractylodes Macrocephala Koidz), Baishao (Paeoniae Radix Alba), Huanglian (Coptidis Rhizoma), Paojiang (Rhizoma Zingiberis Preparata), Chenpi (Citrus Reticulata), Chantui (Cicadae Periostracum), and Wumei (Mume Fructus). It was created by Professor Li Junxiang combined with his own 40 years of clinical experience and has achieved good results in the treatment of IBS-D. Previous studies have shown that TXACD effectively reduced IBS-D, by relieving abdominal pain and diarrhea [4]. However, the underlying mechanism of TXACD in the treatment of IBS-D still remains unclear, and the pharmacodynamic properties of its components and key targets remain to be identified.

**Figure 1:**
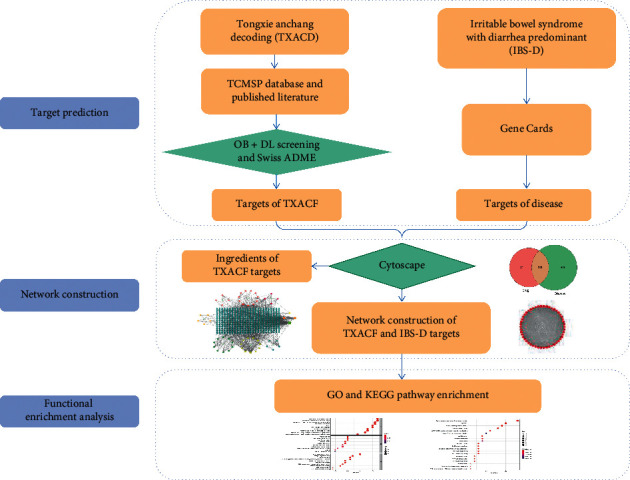
The workflow of the network pharmacological study of TXACD in the treatment of IBS-D.

Network pharmacology is a novel method based on system biology and multidirectional pharmacology. It combines computer biology and network analysis to explain the interaction among drugs, targets, and diseases from the point of view of multicomponents, multitargets, and multipathways [5]. The integrity and systematicness of the research strategy of network pharmacology is consistent with the overall view of TCM, which provides a method for the study of the action mechanism of multicomponent TCM [6–8]. Therefore, this study aims to use the method of network pharmacology to explore the molecular mechanism of TXACD in the treatment of IBS-D, in order to provide a basis for further research. The workflow of this study is shown in Figure 1.

## 2. Materials and Methods

### 2.1. Chemical Database Collection and Construction

TCMSP (http://tcmspw.com/tcmsp.php) [9] and published literature were used to collect the active compounds in TXACD. Oral bioavailability (OB) and drug-like (DL) in TCMSP were chosen as screening parameters for the chemical composition of TCM. Oral bioavailability indicates the relative amount of orally administered drug that reaches the blood circulation. Drug-like is a qualitative concept, indicating the similarity of a compound with a known drug [10]. The compounds conforming to the requirements of both OB ≥ 30% and DL ≥ 0.18 were selected as active compounds. We screened the related compounds of herbs from published literature and got the corresponding CAS number from ChemSrc database (https://www.chemsrc.com). Then, the CAS number was imported into PubChem database (http://pubchem.ncbi.nlm.nih.gov) [11] to obtain the corresponding 2D structure of compounds. And SwissADME (http://www.swissadme.ch) [12] was used to screen the compounds in TXACD based on the 2D structure. SwissADME is a website that can compute physicochemical descriptors as well as predict ADME parameters, pharmacokinetic properties, and drug-like nature. Also, those compounds without target information were excluded.

### 2.2. Identified and Predicted Targets of TXACD

The TCMSP and Swiss Target Prediction (http://www.swisstargetprediction.ch) [13] were used to identify the potential target genes of the active compounds in TXACD. The gene information, including the name, gene symbol, and organism, was confirmed using the UniProt protein sequence resource (https://www.uniprot.org) [14]. The active compounds without targets and repeated targets were removed.

### 2.3. Construction of Active Compound-Target Network

The active compound-target network was constructed by the Cytoscape software (version 3.6.1). The identified active compounds and potential targets were imported to Cytoscape software. The active compound-target network reflects the important relationship between active compounds and potential targets. Nodes represent the compounds and targets, while edges indicate the intermolecular interactions between compounds and targets. By using the network analyzer tool to analyze the active compound-target network, the degree value of the node can be calculated. The larger the value is, the more likely the compound is to become the key compound of herb.

### 2.4. Targets of IBS-D

Potential targets information related to IBS-D was obtained from the GeneCards database (http://www.genecards.org) [15] which is a comprehensive database of functions, including genomics, proteomics, and transcriptomics. The“ irritable bowel syndrome with diarrhea predominant” was used as the keyword to screen targets. According to the median of relevance score, the disease targets which scored above the median were filtrated for further analysis.

### 2.5. PPI Network Construction

To obtain compound-disease common targets, we integrated the screened active compound targets and disease targets by using the ImageGP platform. The common targets were imported to the STRING database (https://string-db.org/) [16] for the protein interaction analysis, and organism was set as “Homo sapiens”. The result obtained from the STRING database was edited by the Cytoscape software. The plug-in CytoHubba (http://apps.cytoscape.org/apps/cytohubba) was used to screen out the hug genes according to the maximal clique centrality (MCC) of topology analysis, and the top 50 hub genes were used to construct the PPI network. The nodes represent proteins, and the edges reflect the interaction between the proteins.

### 2.6. Gene Ontology and KEGG Pathway Enrichment Analyses

Gene ontology (GO) (http://geneontology.org) is a widely used ontology in the field of bioinformatics, which covers three aspects of biology: biological processes (BP), cellular components (CC), and molecular functions (MF) [17–19]. Kyoto Encyclopedia of Genes and Genomes (KEGG) (https://www.kegg.jp) [20, 21] is a utility database resource for understanding advanced functions and biological systems (such as cells, organisms, and ecosystems), genome sequencing, and other high-throughput experimental techniques generated from molecular level information. The *R* package was used for enrichment analysis based on the STRING database. The threshold was set as *p* adjust value < 0.05.

## 3. Results

### 3.1. Screening for the Active Compounds of TXACD

TCMSP database and published literature were used to search for the active compounds of TXACD. A total of 54 active compounds were obtained in accordance with good ADME properties. There were 8 active compounds of Baishao (BS), 4 active compounds of Baizhu (BZ), 5 active compounds of Chenpi (CP), 11 active compounds of Huanglian (HL), 8 active compounds of Wumei (WM), 11 active compounds of Paojiang (PJ), and 11 active compounds of Chantui (CT). Among these active compounds, beta-sitosterol is a common compound of BS and WM. Sitosterol is a common compound of BS and CP. Kaempferol is a common compound of BS and WM. Quercetin is a common compound of HL and WM. The detailed information of active compounds is described in Supplementary materials 1.

### 3.2. Putative Target Prediction for the Candidate Targets of TXACD

We used the TCMSP and Swiss target prediction databases to screen the targets of TXACD. As a result, 639 targets were obtained (in Supplementary materials 2). Among these targets, there were 116 targets corresponding to BS, 22 targets corresponding to BZ, 90 targets corresponding to CP, 275 targets corresponding to HL, 287 targets corresponding to WM, 227 targets corresponding to CT, and 282 targets corresponding to PJ. GeneCards database was used to screen 855 targets related to IBS-D (in Supplementary materials 3). The active compounds and targets obtained above were used for construction of subsequent networks.

### 3.3. Compound-Target Network Construction

**Figure 2:**
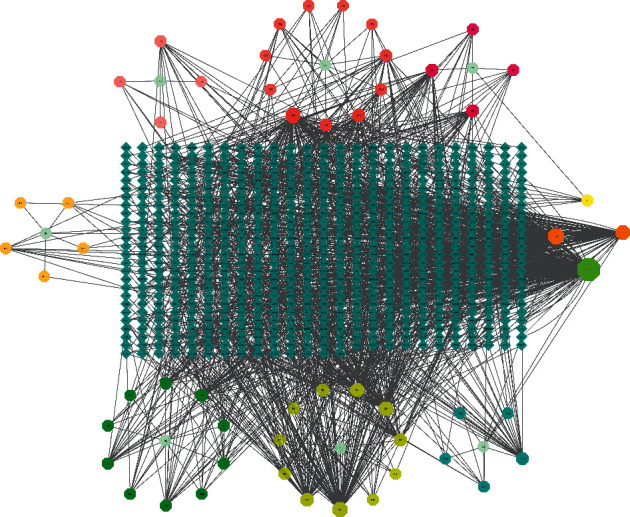
Compound-target network of potential targets in TXACD. The octagon nodes represent the active compounds in TXACD, the diamond nodes represent the corresponding targets of the compounds, and the ellipse nodes represent the herb names.

We constructed a compound-target network to understand the interaction relationship between the compounds and their corresponding targets (Figure 2). This network consisted of 700 nodes (54 compounds, 639 targets, and 7 herb names) and 1457 edges. Based on the network analysis, the compounds with more targets were quercetin (degree = 298), kaempferol (degree = 122), 6-gingerol (degree = 95), (2R,3S)-2-(3',4'-dihydroxyphenyl)-3-acetylamino-7-(*N*-acetyl-2′′-aminoethyl)-1,4-piperocycline (degree = 95), beta-sitosterol (degree = 74), and flavone (degree = 70). The result suggested that these active compounds with high values of degree might serve as significant therapeutic compounds in IBS-D. At the same time, it indicated the multitarget treatment characteristics of TXACD.

### 3.4. Analysis of Hub Gene and Construction of the Protein-Protein Interaction (PPI) Network

**Figure 3:**
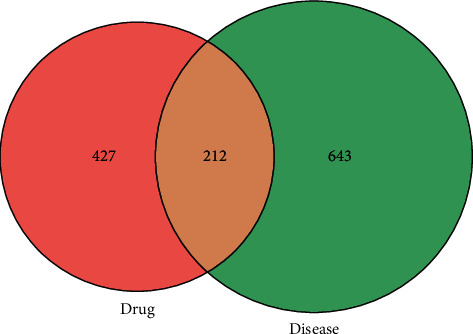
Venn diagram of targets for TXACD treating IBS-D. The pink circle represents the related targets of TXACD. The green circle represents the related targets of IBS-D.

**Figure 4:**
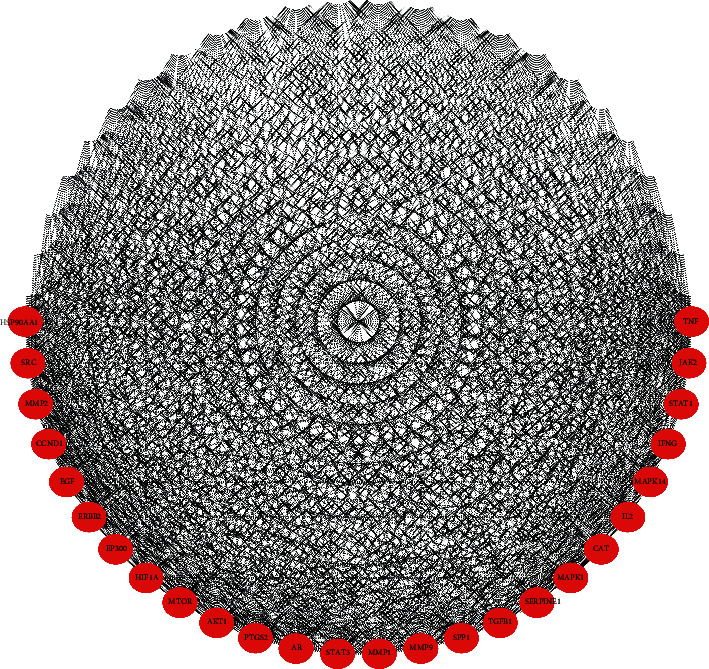
PPI network of targets for TXACD in the treatment of IBS-D.

We screened 639 corresponding targets of the compounds in TXACD and 855 targets related to IBS-D. 212 targets were obtained after taking the intersection of compound targets and disease targets (Figure 3). The obtained targets were the potential targets in the treatment of IBS-D. Then, 212 targets were uploaded to the STRING database, the PPI score was set as a medium confidence of 0.400, and the active interaction of proteins was chosen from databases. The results were imported into Cytoscape software for further analysis. The top 50 hub genes were obtained based on the MCC method of plug-in CytoHubba, including VEGFA, MAPK3, IL6, TP53, JUN, STAT3, TNF, PTGS2, MAPK1, CASP3, MAPK8, MMP9, MYC, EGFR, EGF, SRC, BCL2L1, CXCL8, MTOR, CCND1, PTEN, MAP2K1, AKT1, IL10, MMP2, MAPK14, CAT, ASP9, FOS, ESR1, CREB1, CASP8, IL2, HSP90AA1, CCL2, ERBB2, STAT1, CDKN1A, JAK2, IL1B, IGF1R, ICAM1, EP300, HIF1A, TGFB1, MMP1, SERPINE1, SPP1, IFNG, and AR. Hub gene as an important target plays a crucial role in biological processes [22]. In related pathways, the regulation of other genes is often affected by the hub gene. Then, the hub genes were used for construction of PPI network which consisted of 50 nodes and 269 edges. The results were used for further analysis through Cytoscape software, and the network was constructed as Figure 4.

### 3.5. GO and KEGG Pathway Enrichment Analyses

**Figure 5:**
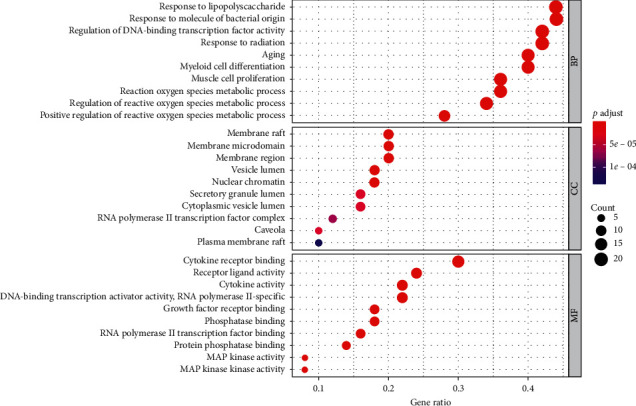
Enriched GO terms for biological process (BP), cellular component (CC), and molecular function (MF).

The hub genes were imported into the STRING database for GO and KEGG pathway enrichment analyses. The complete results of the GO enrichment analysis are displayed in Supplementary materials 4. The top ten results for the three aspects are shown in Figure 5. These targets of biological processes (BP) mainly involved response to lipopolysaccharide, response to molecule of bacterial origin, regulation of DNA-binding transcription factor activity, response to radiation, aging, myeloid cell differentiation, muscle cell proliferation, and reactive oxygen species metabolic process, regulation of reactive oxygen species metabolic process, and positive regulation of reactive oxygen species metabolic process. Based on cell component (CC) analysis, the targets were mainly related to membrane raft, membrane microdomain, membrane region, vesicle lumen, nuclear chromatin, secretory granule lumen, cytoplasmic vesicle lumen, RNA polymerase II transcription factor complex, caveola, and plasma membrane raft. According to the molecular function (MF) analysis, the targets were closely related to cytokine receptor binding, receptor ligand activity, cytokine activity, DNA-binding transcription activator activity, RNA polymerase II-specific, growth factor receptor binding, phosphatase binding, RNA polymerase II transcription factor binding, protein phosphatase binding, MAP kinase activity, and MAP kinase kinase activity.

**Figure 6:**
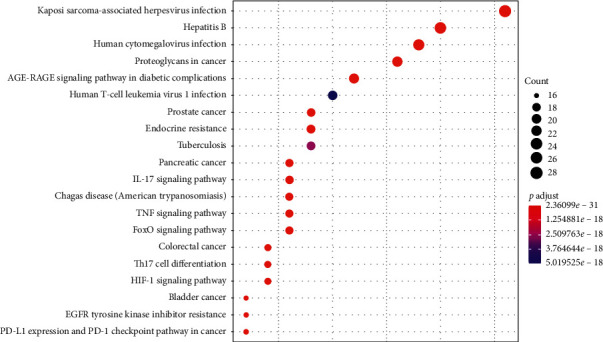
Enriched terms in KEGG pathway enrichment analysis.

The results of the KEGG analysis are displayed in supplementary materials 5.  Figure 6 shows the 20 most prominent signal pathways. KEGG pathway enrichment analysis indicated that the target genes were involved in numerous pathways, such as IL-17 signaling pathway, TNF signaling pathway, Th17 cell differentiation, and HIF-1 signaling pathway which are closely associated with immune regulation, inflammatory response, and oxidative stress. FoxO signaling pathway is related to signal transduction. Proteoglycans, prostate cancer, pancreatic cancer, and bladder cancer play an important part in pathways in cancer. Moreover, some other pathways such as Kaposi sarcoma-associated herpesvirus infection, hepatitis B, human cytomegalovirus infection, AGE-RAGE signaling pathway in diabetic complications, human T-cell leukemia virus 1 infection, endocrine resistance, tuberculosis, Chagas disease (American trypanosomiasis), EGFR tyrosine kinase inhibitor resistance, PD-L1 expression, and PD-1 checkpoint pathway in cancer were identified. The results suggested that TXACD alleviated IBS-D by regulating multiple pathways.

## 4. Discussion

Irritable bowel syndrome with diarrhea predominant (IBS-D) is one of the most common gastrointestinal disorders, affecting the quality of life for patient and lacking effective therapies [23]. Traditional Chinese medicines (TCMs) with multiple targets and pathways have been shown to treat IBS-D. The previous study of Tongxie Anchang Formula (TXACD) created by Professor Li Junxiang has shown that TXACD effectively alleviated IBS-D. However, the underlying mechanisms of TXACD in the treatment of IBS-D still remain unknown. In this study, we used a method of network pharmacology to determine the possible mechanisms of TXACD in IBS-D.

As we can see from the compound-target network, each active compound in the network is connected with many targets. It suggested that TXACD has the biological characteristics of multicomponent and multitarget in treating IBS-D. Quercetin (degree = 298), kaempferol (degree = 122), 6-gingerol (degree = 95), (2R,3S)-2-(3′,4′-dihydroxyphenyl)-3-acetylamino-7-(*N*-acetyl-2′′-aminoethyl)-1,4-piperocycline (degree = 95), beta-sitosterol (degree = 74), and flavone (degree = 70) are the main active compounds of TXACD, demonstrating that they played an crucial role in the treatment of IBS-D. It was reported that quercetin could attenuate oxidative stress and DNA damage by regulating NRF2/Keap1 signaling pathway [24] and reverse cell damage induced by H_2_O_2_ [25]. Kaempferol, as a flavonol, has the function of anti-inflammation and antioxidative stress by regulating the SIRT1/HMGB1/NF-kB axis [26, 27]. 6-Gingerol has been reported to promote gut health via suppression of oxidoinflammatory stress responses [28] and improving mitochondrial functions [29].

We constructed the PPI network of hub genes of 212 common targets in the active compound and IBS-D targets. In these targets, VEGFA, MAPK3, IL-6, TP53, and JUN were regarded as significant. VEGFA is a representative *β*-catenin target gene which interacts with adhesion molecules. It was reported that VEGFA was closely related to chronic inflammation [30]. SOFI et al. [31] found that ancient wheat products could ameliorate the severity of gastrointestinal symptoms of IBS by reducing in the circulating levels of VEGFA and IL-6. MAPK3, known as extracellular-regulated protein kinase 1 (ERK1), plays an important role in regulating pathways such as mediating inflammation, cell survival, cell death, proliferation, and differentiation [32, 33]. IL-6 is a proinflammatory cytokine. Bashashati et al. [34] found that IL-6 level was significantly higher in IBS-D compared to healthy people. It was reported that IL-6 could directly affect gastrointestinal movement [35]. A study was conducted about vitamin D3 supplementation in the treatment of IBS-D, indicating that vitamin D3 supplementation could improve symptoms by modulating the serum level of IL-6 [36]. TP53 and JUN are associated with the regulation of cell proliferation differentiation and apoptosis [37, 38]. Therefore, they play a crucial role in maintaining genetic stability. The p53 protein is characterized as a stress-response molecule [39]. It was reported that a higher proportion of TP53-expressing cells was observed in the most inflamed samples of inflammatory bowel disease (IBD) [40]. JUN, also named c-Jun, is the most potent transcriptional activator of the AP-1 family [41]. Brain-derived neurotrophic factor (BNDF) has been found that it increased in the colonic mucosa of patients with IBS, and interleukin-1*β* could regulate the expression of BNDF via a phosphorylated-c-Jun *N*-terminal kinase pathway [42]. In the situation of intestinal infection, JNK inhibition resulted in reductions of c-Jun expression and significant suppression of proinflammatory cytokines such as IL-1*β*, IL-6, and TNF-*α* [43].

For further study, we performed gene ontology (GO) enrichment for hub genes to predict the underlying mechanism of TXACD in the treatment of IBS-D. GO covers three aspects of biology: biological processes (BP), cellular components (CC), and molecular functions (MF). Based on BP analysis, it could be seen that the targets are associated with oxidative stress, lipopolysaccharide, and other biological processes. Oxidative stress is caused by the excessive generation of reactive oxygen species (ROS) and generates metabolic imbalances that bring about some harmful changes such as inflammation and oxidative tissue damage [44]. Balmus et al. reported that oxidative stress and inflammation were of great importance in IBS pathological development [45]. Mete et al. [46] found that, in IBS patients, malondialdehyde (MDA) and nitric oxide (NO) concentrations were significantly higher than in controls. Meanwhile, Hajizadeh Maleki et al. [47] showed that low-to-moderate intensity exercise training program attenuated symptoms in IBS by reducing the level of MDA and NO. CC analysis indicated that the targets mainly involved membrane raft, membrane microdomain, membrane region, and other cellular components. This indirectly demonstrated the complexity of the pathogenesis of IBS-D and the damage to various cellular components. In addition, MF analysis contained cytokine receptor binding, receptor ligand activity, cytokine activity, and other molecular functions. This result illustrated that the targets influenced the pathological development of IBS-D with diverse molecular functions.

Based on the KEGG pathway enrichment analysis, the potential targets for TXACD in the treatment of IBS-D were mainly related to IL-17 signaling pathway, TNF signaling pathway, Th17 cell differentiation, and HIF-1 signaling pathway. These pathways are closely associated with immune regulation, inflammatory response, and oxidative stress. The cytokines and related pathways of interleukin 17 (IL-17) family play an important role in inflammatory and autoimmune diseases. IL-17, secreted by Th17 cells, mediates the production of other proinflammatory cytokines and chemokines, including TNF-*α*, IL-8, and GM-CSF [48]. Furthermore, Berg et al. [49] reported that compared to controls, TNF, IL-17, and GM-CSF were significantly increased in IBS. These cytokines recruit neutrophils to participate in the development of inflammation. During the inflammatory process, released interleukins can result in an impairment in the cellular antioxidant system and lead to a rapid increase in the production of ROS, which activates the inflammasome and causes inflammation [50]. As mentioned above, oxidative stress and inflammation were of great importance in IBS pathological development, and Wang et al. [51] found that kaempferol had the function of inhibiting oxidative and inflammatory stress. In addition, hypoxia is associated with intestinal inflammation and underlies the polarization of inflammatory T cells such as type 1 T helper (Th1) and Th17 cells in inflamed tissues [52]. Hypoxia-inducible factor-1*α* (HIF-1*α*) is strongly expressed in T cells that infiltrate the inflamed mucosa and plays an important role in the process of inflammation [53]. A recent research suggested that hydroxylase inhibitors, targeting HIF pathway, might be a potential treatment of inflammatory diseases [54].

## 5. Conclusions

In summary, this study aimed to analyze the underlying mechanism of TXACD in IBS-D based on network pharmacology. The results demonstrated that TXACD alleviated IBS-D through multiple pathways, including the immune regulation, inflammatory response, and oxidative stress. In addition, our work has a great value to provide theoretical basis for the treatment of diseases with TCM. However, there are drawbacks to this research as it was based on data mining and analysis. Further clinical trials and verification studies should be carried out on the role of TXACD in IBS-D.

## Data Availability

The data used to support the findings of this study are included within the supplemental files.

## Supplementary Materials

Supplementary Materials Supplementary materials 1: the active compounds of TXACD. Supplementary materials 2: the information on potential targets of TXACD. Supplementary materials 3: the information on potential targets of IBS-D. Supplementary materials 4: raw data of GO enrichment analysis. Supplementary materials 5: raw data of KEGG enrichment analysis.
