# Supplementary material for: Network Pharmacology Identifies the Mechanisms of Action of Tongxie Anchang Decoction in the Treatment of Irritable Bowel Syndrome with Diarrhea Predominant
Source: Evid Based Complement Alternat Med. 2020 Nov 17;2020:2723705. doi: 10.1155/2020/2723705 (PMC7685835; doi:10.1155/2020/2723705)
Supplement: Supplementary Materials — 1: the active compounds of TXACD. Supplementary materials 2: the information on potential targets of TXACD. Supplementary materials 3: the information on potential targets of IBS-D. Supplementary materials 4: raw data of GO enrichment analysis. Supplementary materials 5: raw data of KEGG enrichment analysis. [file 2723705.f1.zip › supplementary material 1.docx]

| Herb | No | Molecule Name | OB(%) | DL | Degree |
| --- | --- | --- | --- | --- | --- |
| BS | BS1 | paeoniflorgenone | 87.59 | 0.37 | 2 |
| BS | BS2 | (3S,5R,8R,9R,10S,14S)-3,17-dihydroxy-4,4,8,10,14-pentamethyl-2,3,5,6,7,9-hexahydro-1H-cyclopenta[a]phenanthrene-15,16-dione | 43.56 | 0.53 | 3 |
| BS | BS3 | paeoniflorin | 53.87 | 0.79 | 5 |
| BS | BS4 | Mairin | 55.38 | 0.78 | 2 |
| BS | B1 | beta-sitosterol | 36.91 | 0.75 | 74 |
| BS | A1 | sitosterol | 36.91 | 0.75 | 8 |
| BS | B2 | kaempferol | 41.88 | 0.24 | 122 |
| BS | BS5 | (+)-catechin | 54.83 | 0.24 | 10 |
| BZ | BZ1 | 14-acetyl-12-senecioyl-2E,8Z,10E-atractylentriol | 63.37 | 0.3 | 2 |
| BZ | BZ2 | (3S,8S,9S,10R,13R,14S,17R)-10,13-dimethyl-17-[(2R,5S)-5-propan-2-yloctan-2-yl]-2,3,4,7,8,9,11,12,14,15,16,17-dodecahydro-1H-cyclopenta[a]phenanthren-3-ol | 36.23 | 0.78 | 2 |
| BZ | BZ3 | 3β-acetoxyatractylone | 54.07 | 0.22 | 16 |
| BZ | BZ4 | 8β-ethoxy atractylenolide Ⅲ | 35.95 | 0.21 | 6 |
| CP | A1 | sitosterol | 36.91 | 0.75 | 8 |
| CP | CP1 | naringenin | 59.29 | 0.21 | 36 |
| CP | CP2 | 5,7-dihydroxy-2-(3-hydroxy-4-methoxyphenyl)chroman-4-one | 47.74 | 0.27 | 10 |
| CP | CP3 | Citromitin | 86.9 | 0.51 | 11 |
| CP | CP4 | nobiletin | 61.67 | 0.52 | 34 |
| HL | HL1 | berberine | 36.86 | 0.78 | 17 |
| HL | HL2 | berberrubine | 35.74 | 0.73 | 13 |
| HL | HL3 | epiberberine | 43.09 | 0.78 | 11 |
| HL | HL4 | (R)-Canadine | 55.37 | 0.77 | 31 |
| HL | HL5 | Berlambine | 36.68 | 0.82 | 20 |
| HL | HL6 | Corchoroside A_qt | 104.95 | 0.78 | 3 |
| HL | HL7 | Magnograndiolide | 63.71 | 0.19 | 5 |
| HL | HL8 | palmatine | 64.6 | 0.65 | 19 |
| HL | C1 | quercetin | 46.43 | 0.28 | 298 |
| HL | HL9 | coptisine | 30.67 | 0.86 | 10 |
| HL | HL10 | Worenine | 45.83 | 0.87 | 8 |
| WM | WM1 | (2R)-5,7-dihydroxy-2-(4-hydroxyphenyl)chroman-4-one | 42.36 | 0.21 | 8 |
| WM | B1 | beta-sitosterol | 36.91 | 0.75 | 74 |
| WM | B2 | kaempferol | 41.88 | 0.24 | 122 |
| WM | WM2 | Stigmasterol | 43.83 | 0.76 | 31 |
| WM | WM3 | campest-5-en-3beta-ol | 37.58 | 0.71 | 2 |
| WM | WM4 | Methyl arachidonate | 46.9 | 0.23 | 3 |
| WM | WM5 | CLR | 37.87 | 0.68 | 4 |
| WM | C1 | quercetin | 46.43 | 0.28 | 298 |

Supplementary material 1 Active compounds and ADME parameters of Tongxie Anchang Decoction (TXACD) screened by TCMSP,Baishao (BS), Baizhu (BZ), Chenpi (CP), Huanglian (HL), Wumei(WM)

A1: the common component of BS and CP; B1: the common component of BS and WM; B2: the common component of BS and WM; C1: the common component of HL and WM

Active compounds and ADME parameters of Tongxie Anchang Decoction (TXACD) screened by swissADME,Paojiang(PJ),Chantui(CT)

| Herb | No | Molecule Name | Degree |
| --- | --- | --- | --- |
| PJ | PJ1 | 6-Gingerol | 95 |
| PJ | PJ2 | 8-Gingerol | 34 |
| PJ | PJ3 | Flavone | 70 |
| PJ | PJ4 | 3-methyloct-1-yn-3-ol | 59 |
| PJ | PJ5 | zingerone | 33 |
| PJ | PJ6 | (+)-borneol | 13 |
| PJ | PJ7 | Decanol | 3 |
| PJ | PJ8 | 10-Gingerol | 21 |
| PJ | PJ9 | 6-Shogaol | 50 |
| PJ | PJ10 | FARNESAL | 10 |
| PJ | PJ11 | Cyclohexane-1,4-dimethanol | 5 |
| CT | CT1 | 4-Aminobutyricacid | 26 |
| CT | CT2 | Phenylalanine | 25 |
| CT | CT3 | L-Methionine | 6 |
| CT | CT4 | L-glutamicacid | 6 |
| CT | CT5 | L-Lysine | 6 |
| CT | CT6 | Tyrosine | 11 |
| CT | CT7 | l-asparticacid | 2 |
| CT | CT8 | [Ranachrome4](https://www.chemsrc.com/cas/529-69-1_122008.html) | 4 |
| CT | CT9 | (2R,3S)-2-(3',4'-dihydroxyphenyl)-3-acetylamino-7-(N-acetyl-2″-aminoethyl)-1,4-piperocycline | 94 |
| CT | CT10 | (2R,3S)-2-(3',4'-dihydroxyphenyl)-3-acetylamino-6-(N-acetyl-2″-aminoethyl)-1,4-piperocycline | 42 |
| CT | CT11 | (2R,3S)-2-(3',4'-dihydroxyphenyl)-3-acetylamino-7-(n-acetyl-2″-aminovinyl)-1,4-piperoxolane | 16 |
