# Supplementary material for: Network Pharmacology Identifies the Mechanisms of Action of Tongxie Anchang Decoction in the Treatment of Irritable Bowel Syndrome with Diarrhea Predominant
Source: Evid Based Complement Alternat Med. 2020 Nov 17;2020:2723705. doi: 10.1155/2020/2723705 (PMC7685835; doi:10.1155/2020/2723705)
Supplement: Supplementary Materials — 1: the active compounds of TXACD. Supplementary materials 2: the information on potential targets of TXACD. Supplementary materials 3: the information on potential targets of IBS-D. Supplementary materials 4: raw data of GO enrichment analysis. Supplementary materials 5: raw data of KEGG enrichment analysis. [file 2723705.f1.zip › supplementary material 2.docx]

Supplementary material 2 Information on potential targets of TXACD

| No. | Gene name | Protein name | UniProt ID | Organism |
| --- | --- | --- | --- | --- |
| 1 | GABRA1 | Gamma-aminobutyric acid receptor subunit alpha-1 | P14867 | Homo sapiens |
| 2 | PGR | Progesterone receptor | P06401 | Homo sapiens |
| 3 | NR3C2 | Mineralocorticoid receptor | P08235 | Homo sapiens |
| 4 | TNF | Tumor necrosis factor | P01375 | Homo sapiens |
| 5 | IL6 | Interleukin-6 | P05231 | Homo sapiens |
| 6 | CD14 | Monocyte differentiation antigen CD14 | P08571 | Homo sapiens |
| 7 | LBP | Lipopolysaccharide-binding protein | P18428 | Homo sapiens |
| 8 | NCOA2 | Nuclear receptor coactivator 2 | Q15596 | Homo sapiens |
| 9 | PTGS1 | Prostaglandin G/H synthase 1 | P23219 | Homo sapiens |
| 10 | PTGS2 | Prostaglandin G/H synthase 2 | P35354 | Homo sapiens |
| 11 | HSP90AB1 | Heat shock protein HSP 90 | P08238 | Homo sapiens |
| 12 | PIK3CG | Phosphatidylinositol-4,5-bisphosphate 3-kinase catalytic subunit, gamma isoform | P48736 | Homo sapiens |
| 13 | KCNH2 | Potassium voltage-gated channel subfamily H member 2 | Q12809 | Homo sapiens |
| 14 | DRD1 | Dopamine D1 receptor | P21728 | Homo sapiens |
| 15 | CHRM3 | Muscarinic acetylcholine receptor M3 | P20309 | Homo sapiens |
| 16 | CHRM1 | Muscarinic acetylcholine receptor M1 | P11229 | Homo sapiens |
| 17 | SCN5A | Sodium channel protein type 5 subunit alpha | Q14524 | Homo sapiens |
| 18 | GABRA2 | Gamma-aminobutyric-acid receptor alpha-2 subunit | P47869 | Homo sapiens |
| 19 | CHRM4 | Muscarinic acetylcholine receptor M4 | P08173 | Homo sapiens |
| 20 | PDE3A | CGMP-inhibited 3',5'-cyclic phosphodiesterase A | Q14432 | Homo sapiens |
| 21 | HTR2A | 5-hydroxytryptamine 2A receptor | P28223 | Homo sapiens |
| 22 | GABRA5 | Gamma-aminobutyric-acid receptor alpha-5 subunit | P31644 | Homo sapiens |
| 23 | ADRA1A | Alpha-1A adrenergic receptor | P35348 | Homo sapiens |
| 24 | GABRA3 | Gamma-aminobutyric-acid receptor alpha-3 subunit | P34903 | Homo sapiens |
| 25 | CHRM2 | Muscarinic acetylcholine receptor M2 | P08172 | Homo sapiens |
| 26 | ADRA1B | Alpha-1B adrenergic receptor | P35368 | Homo sapiens |
| 27 | ADRB2 | Beta-2 adrenergic receptor | P07550 | Homo sapiens |
| 28 | CHRNA2 | Neuronal acetylcholine receptor subunit alpha-2 | Q15822 | Homo sapiens |
| 29 | SLC6A4 | Sodium-dependent serotonin transporter | P31645 | Homo sapiens |
| 30 | OPRM1 | Mu-type opioid receptor | P35372 | Homo sapiens |
| 31 | CHRNA7 | Neuronal acetylcholine receptor protein, alpha-7 chain | P36544 | Homo sapiens |
| 32 | BCL2 | Apoptosis regulator Bcl-2 | P10415 | Homo sapiens |
| 33 | BAX | Apoptosis regulator BAX | Q07812 | Homo sapiens |
| 34 | CASP9 | Caspase-9 | P55211 | Homo sapiens |
| 35 | JUN | Transcription factor AP-1 | P05412 | Homo sapiens |
| 36 | CASP3 | Caspase-3 | P42574 | Homo sapiens |
| 37 | CASP8 | Caspase-8 | Q14790 | Homo sapiens |
| 38 | PRKCA | Protein kinase C alpha type | P17252 | Homo sapiens |
| 39 | TGFB1 | Transforming growth factor beta-1 | P01137 | Homo sapiens |
| 40 | PON1 | Serum paraoxonase/arylesterase 1 | P27169 | Homo sapiens |
| 41 | MAP2 | Microtubule-associated protein 2 | P11137 | Homo sapiens |
| 42 | NOS2 | Nitric oxide synthase, inducible | P35228 | Homo sapiens |
| 43 | AR | Androgen receptor | P10275 | Homo sapiens |
| 44 | PPARG | Peroxisome proliferator activated receptor gamma | P37231 | Homo sapiens |
| 45 | DPP4 | Dipeptidyl peptidase IV | P27487 | Homo sapiens |
| 46 | PRSS1 | Trypsin-1 | P07477 | Homo sapiens |
| 47 | NOS3 | Nitric-oxide synthase, endothelial | P29474 | Homo sapiens |
| 48 | ACHE | Acetylcholinesterase | P22303 | Homo sapiens |
| 49 | SLC6A2 | Sodium-dependent noradrenaline transporter | P23975 | Homo sapiens |
| 50 | TOP2A | DNA topoisomerase II | P11388 | Homo sapiens |
| 51 | F7 | Coagulation factor VII | P08709 | Homo sapiens |
| 52 | Calmodulin-1 | Calmodulin | P0DP23 | Homo sapiens |
| 53 | RELA | Transcription factor p65 | Q04206 | Homo sapiens |
| 54 | IKBKB | Inhibitor of nuclear factor kappa-B kinase subunit beta | O14920 | Homo sapiens |
| 55 | AKT1 | RAC-alpha serine/threonine-protein kinase | P31749 | Homo sapiens |
| 56 | AHSA1 | Activator of 90 kDa heat shock protein ATPase homolog 1 | O95433 | Homo sapiens |
| 57 | MAPK8 | Mitogen-activated protein kinase 8 | P45983 | Homo sapiens |
| 58 | XDH | Xanthine dehydrogenase/oxidase | P47989 | Homo sapiens |
| 59 | MMP1 | Interstitial collagenase | P03956 | Homo sapiens |
| 60 | STAT1 | Signal transducer and activator of transcription 1-alpha/beta | P42224 | Homo sapiens |
| 61 | CDK1 | Cell division control protein 2 homolog | P06493 | Homo sapiens |
| 62 | HMOX1 | Heme oxygenase 1 | P09601 | Homo sapiens |
| 63 | CYP3A4 | Cytochrome P450 3A4 | P08684 | Homo sapiens |
| 64 | CYP1A2 | Cytochrome P450 1A2 | P05177 | Homo sapiens |
| 65 | CYP1A1 | Cytochrome P450 1A1 | P04798 | Homo sapiens |
| 66 | ICAM1 | Intercellular adhesion molecule 1 | P05362 | Homo sapiens |
| 67 | SELE | E-selectin | P16581 | Homo sapiens |
| 68 | VCAM1 | Vascular cell adhesion protein 1 | P19320 | Homo sapiens |
| 69 | NR1I2 | Nuclear receptor subfamily 1 group I member 2 | O75469 | Homo sapiens |
| 70 | CYP1B1 | Cytochrome P450 1B1 | Q16678 | Homo sapiens |
| 71 | ALOX5 | Arachidonate 5-lipoxygenase | P09917 | Homo sapiens |
| 72 | HAS2 | Hyaluronan synthase 2 | Q92819 | Homo sapiens |
| 73 | GSTP1 | Glutathione S-transferase P | P09211 | Homo sapiens |
| 74 | AHR | Aryl hydrocarbon receptor | P35869 | Homo sapiens |
| 75 | PSMD3 | 26S proteasome non-ATPase regulatory subunit 3 | O43242 | Homo sapiens |
| 76 | SLC2A4 | Solute carrier family 2, facilitated glucose transporter member 4 | P14672 | Homo sapiens |
| 77 | NR1I3 | Nuclear receptor subfamily 1 group I member 3 | Q14994 | Homo sapiens |
| 78 | INSR | Insulin receptor | P06213 | Homo sapiens |
| 79 | DIO1 | Type I iodothyronine deiodinase | P49895 | Homo sapiens |
| 80 | PPP3CA | Serine/threonine-protein phosphatase 2B catalytic subunit alpha isoform | Q08209 | Homo sapiens |
| 81 | GSTM1 | Glutathione S-transferase Mu 1 | P09488 | Homo sapiens |
| 82 | GSTM2 | Glutathione S-transferase Mu 2 | P28161 | Homo sapiens |
| 83 | AKR1C3 | Aldo-keto reductase family 1 member C3 | P42330 | Homo sapiens |
| 84 | SLPI | Antileukoproteinase | P03973 | Homo sapiens |
| 85 | ESR1 | Estrogen receptor | P03372 | Homo sapiens |
| 86 | RXRA | Retinoic acid receptor RXR-alpha | P19793 | Homo sapiens |
| 87 | CAT | Catalase | P04040 | Homo sapiens |
| 88 | NCOA1 | Nuclear receptor coactivator 1 | Q15788 | Homo sapiens |
| 89 | MAPK3 | Mitogen-activated protein kinase 3 | P27361 | Homo sapiens |
| 90 | MAPK1 | Mitogen-activated protein kinase 1 | P28482 | Homo sapiens |
| 91 | FASN | Fatty acid synthase | P49327 | Homo sapiens |
| 92 | LDLR | Low-density lipoprotein receptor | P01130 | Homo sapiens |
| 93 | BAD | Bcl2 antagonist of cell death | Q92934 | Homo sapiens |
| 94 | SOD1 | Superoxide dismutase [Cu-Zn] | P00441 | Homo sapiens |
| 95 | MTTP | Microsomal triglyceride transfer protein large subunit | P55157 | Homo sapiens |
| 96 | APOB | Apolipoprotein B-100 | P04114 | Homo sapiens |
| 97 | PLB1 | Phospholipase B1, membrane-associated | Q6P1J6 | Homo sapiens |
| 98 | HMGCR | 3-hydroxy-3-methylglutaryl-coenzyme A reductase | P04035 | Homo sapiens |
| 99 | CYP19A1 | Cytochrome P450 19A1 | P11511 | Homo sapiens |
| 100 | UGT1A1 | UDP-glucuronosyltransferase 1-1 | P22309 | Homo sapiens |
| 101 | PPARA | Peroxisome proliferator-activated receptor alpha | Q07869 | Homo sapiens |
| 102 | SREBF1 | Sterol regulatory element-binding protein 1 | P36956 | Homo sapiens |
| 103 | GSR | Glutathione reductase, mitochondrial | P00390 | Homo sapiens |
| 104 | ABCC1 | Multidrug resistance-associated protein 1 | P33527 | Homo sapiens |
| 105 | ADIPOQ | Adiponectin | Q15848 | Homo sapiens |
| 106 | SOAT2 | Sterol O-acyltransferase 2 | O75908 | Homo sapiens |
| 107 | AKR1C1 | Aldo-keto reductase family 1 member C1 | Q04828 | Homo sapiens |
| 108 | GOT1 | Aspartate aminotransferase, cytoplasmic | P17174 | Homo sapiens |
| 109 | ABAT | 4-aminobutyrate aminotransferase, mitochondrial | P80404 | Homo sapiens |
| 110 | CES1 | Liver carboxylesterase 1 | P23141 | Homo sapiens |
| 111 | SOAT1 | Sterol O-acyltransferase 1 | P35610 | Homo sapiens |
| 112 | TOP2A | DNA topoisomerase II | P11388 | Homo sapiens |
| 113 | F10 | Coagulation factor Xa | P00742 | Homo sapiens |
| 114 | KCNMA1 | Calcium-activated potassium channel subunit alpha 1 | Q12791 | Homo sapiens |
| 115 | ESR2 | Estrogen receptor beta | Q92731 | Homo sapiens |
| 116 | CHEK1 | Serine/threonine-protein kinase Chk1 | O14757 | Homo sapiens |
| 117 | GSK3B | Glycogen synthase kinase-3 beta | P49841 | Homo sapiens |
| 118 | MMP9 | Matrix metalloproteinase-9 | P14780 | Homo sapiens |
| 119 | TP53 | Cellular tumor antigen p53 | P04637 | Homo sapiens |
| 120 | TIMP1 | Metalloproteinase inhibitor 1 | P01033 | Homo sapiens |
| 121 | CREB1 | Cyclic AMP-responsive element-binding protein 1 | P16220 | Homo sapiens |
| 122 | PLA2G4A | Cytosolic phospholipase A2 | P47712 | Homo sapiens |
| 123 | CD163 | Scavenger receptor cysteine-rich type 1 protein M130 | Q86VB7 | Homo sapiens |
| 124 | EPHB2 | Ephrin type-B receptor 2 | P29323 | Homo sapiens |
| 125 | PDE10A | cAMP and cAMP-inhibited cGMP 3',5'-cyclic phosphodiesterase 10A | Q9Y233 | Homo sapiens |
| 126 | CHRM5 | Muscarinic acetylcholine receptor M5 | P08912 | Homo sapiens |
| 127 | HTR3A | 5-hydroxytryptamine receptor 3A | P46098 | Homo sapiens |
| 128 | ADRA2C | Alpha-2C adrenergic receptor | P18825 | Homo sapiens |
| 129 | OPRD1 | Delta-type opioid receptor | P41143 | Homo sapiens |
| 130 | HTR2C | 5-hydroxytryptamine 2C receptor | P28335 | Homo sapiens |
| 131 | SLC6A3 | Sodium-dependent dopamine transporter | Q01959 | Homo sapiens |
| 132 | ADRA1D | Alpha-1D adrenergic receptor | P25100 | Homo sapiens |
| 133 | DRD5 | D(1B) dopamine receptor | P21918 | Homo sapiens |
| 134 | GRIA2 | Glutamate receptor 2 | P42262 | Homo sapiens |
| 135 | GABRA6 | Gamma-aminobutyric-acid receptor subunit alpha-6 | Q16445 | Homo sapiens |
| 136 | PIM1 | Proto-oncogene serine/threonine-protein kinase Pim-1 | P11309 | Homo sapiens |
| 137 | CDK2 | Cell division protein kinase 2 | P24941 | Homo sapiens |
| 138 | AKR1B1 | Aldose reductase | P15121 | Homo sapiens |
| 139 | MMP3 | Stromelysin-1 | P08254 | Homo sapiens |
| 140 | MAOB | Amine oxidase [flavin-containing] B | P27338 | Homo sapiens |
| 141 | EGFR | Epidermal growth factor receptor | P00533 | Homo sapiens |
| 142 | VEGFA | Vascular endothelial growth factor A | P15692 | Homo sapiens |
| 143 | CCND1 | G1/S-specific cyclin-D1 | P24385 | Homo sapiens |
| 144 | BCL2L1 | Bcl-2-like protein 1 | Q07817 | Homo sapiens |
| 145 | FOS | Proto-oncogene c-Fos | P01100 | Homo sapiens |
| 146 | CDKN1A | Cyclin-dependent kinase inhibitor 1 | P38936 | Homo sapiens |
| 147 | EIF6 | Eukaryotic translation initiation factor 6 | P56537 | Homo sapiens |
| 148 | PLAU | Urokinase-type plasminogen activator | P00749 | Homo sapiens |
| 149 | MMP2 | 72 kDa type IV collagenase | P08253 | Homo sapiens |
| 150 | IL10 | Interleukin-10 | P22301 | Homo sapiens |
| 151 | EGF | Pro-epidermal growth factor | P01133 | Homo sapiens |
| 152 | RB1 | Retinoblastoma-associated protein | P06400 | Homo sapiens |
| 153 | ELK1 | ETS domain-containing protein Elk-1 | P19419 | Homo sapiens |
| 154 | NFKBIA | NF-kappa-B inhibitor alpha | P25963 | Homo sapiens |
| 155 | POR | NADPH--cytochrome P450 reductase | P16435 | Homo sapiens |
| 156 | ODC1 | Ornithine decarboxylase | P11926 | Homo sapiens |
| 157 | TOP1 | DNA topoisomerase 1 | P11387 | Homo sapiens |
| 158 | RAF1 | RAF proto-oncogene serine/threonine-protein kinase | P04049 | Homo sapiens |
| 159 | HIF1A | Hypoxia-inducible factor 1-alpha | Q16665 | Homo sapiens |
| 160 | RUNX1T1 | Protein CBFA2T1 | Q06455 | Homo sapiens |
| 161 | HSPA5 | 78 kDa glucose-regulated protein | P11021 | Homo sapiens |
| 162 | ERBB2 | Receptor tyrosine-protein kinase erbB-2 | P04626 | Homo sapiens |
| 163 | ACACA | Acetyl-CoA carboxylase 1 | Q13085 | Homo sapiens |
| 164 | CAV1 | Caveolin-1 | Q03135 | Homo sapiens |
| 165 | MYC | Myc proto-oncogene protein | P01106 | Homo sapiens |
| 166 | F3 | Tissue factor | P13726 | Homo sapiens |
| 167 | GJA1 | Gap junction alpha-1 protein | P17302 | Homo sapiens |
| 168 | IL1B | Interleukin-1 beta | P01584 | Homo sapiens |
| 169 | CCL2 | C-C motif chemokine 2 | P13500 | Homo sapiens |
| 170 | PTGER3 | Prostaglandin E2 receptor EP3 subtype | P43115 | Homo sapiens |
| 171 | CXCL8 | Interleukin-8 | P10145 | Homo sapiens |
| 172 | PRKCB | Protein kinase C beta type | P05771 | Homo sapiens |
| 173 | BIRC5 | Baculoviral IAP repeat-containing protein 5 | O15392 | Homo sapiens |
| 174 | DUOX2 | Dual oxidase 2 | Q9NRD8 | Homo sapiens |
| 175 | HSPB1 | Heat shock protein beta-1 | P04792 | Homo sapiens |
| 176 | SULT1E1 | Estrogen sulfotransferase | P49888 | Homo sapiens |
| 177 | MGAM | Maltase-glucoamylase, intestinal | O43451 | Homo sapiens |
| 178 | IL2 | Interleukin-2 | P60568 | Homo sapiens |
| 179 | CCNB1 | G2/mitotic-specific cyclin-B1 | P14635 | Homo sapiens |
| 180 | PLAT | Tissue-type plasminogen activator | P00750 | Homo sapiens |
| 181 | THBD | Thrombomodulin | P07204 | Homo sapiens |
| 182 | SERPINE1 | Plasminogen activator inhibitor 1 | P05121 | Homo sapiens |
| 183 | COL1A1 | Collagen alpha-1(I) chain | P02452 | Homo sapiens |
| 184 | IFNG | Interferon gamma | P01579 | Homo sapiens |
| 185 | PTEN | Phosphatidylinositol-3,4,5-trisphosphate 3-phosphatase and dual-specificity protein phosphatase PTEN | P60484 | Homo sapiens |
| 186 | IL1A | Interleukin-1 alpha | P01583 | Homo sapiens |
| 187 | MPO | Myeloperoxidase | P05164 | Homo sapiens |
| 188 | NCF1 | Neutrophil cytosol factor 1 | P14598 | Homo sapiens |
| 189 | ABCA2 | ATP-binding cassette sub-family G member 2 | Q9BZC7 | Homo sapiens |
| 190 | NFE2L2 | Nuclear factor erythroid 2-related factor 2 | Q16236 | Homo sapiens |
| 191 | NQO1 | NAD(P)H dehydrogenase [quinone] 1 | P15559 | Homo sapiens |
| 192 | PARP1 | Poly [ADP-ribose] polymerase 1 | P09874 | Homo sapiens |
| 193 | COL3A1 | Collagen alpha-1(III) chain | P02461 | Homo sapiens |
| 194 | CXCL11 | C-X-C motif chemokine 11 | O14625 | Homo sapiens |
| 195 | CXCL2 | C-X-C motif chemokine 2 | P19875 | Homo sapiens |
| 196 | DCAF5 | DDB1- and CUL4-associated factor 5 | Q96JK2 | Homo sapiens |
| 197 | CHEK2 | Serine/threonine-protein kinase Chk2 | O96017 | Homo sapiens |
| 198 | CLDN4 | Claudin-4 | O14493 | Homo sapiens |
| 199 | PPARD | Peroxisome proliferator-activated receptor delta | Q03181 | Homo sapiens |
| 200 | HSF1 | Heat shock factor protein 1 | Q00613 | Homo sapiens |
| 201 | CRP | C-reactive protein | P02741 | Homo sapiens |
| 202 | CXCL10 | C-X-C motif chemokine 10 | P02778 | Homo sapiens |
| 203 | CHUK | Inhibitor of nuclear factor kappa-B kinase subunit alpha | O15111 | Homo sapiens |
| 204 | SPP1 | Osteopontin | P10451 | Homo sapiens |
| 205 | RUNX2 | Runt-related transcription factor 2 | Q13950 | Homo sapiens |
| 206 | RASSF1 | Ras association domain-containing protein 1 | Q9NS23 | Homo sapiens |
| 207 | E2F1 | Transcription factor E2F1 | Q01094 | Homo sapiens |
| 208 | E2F2 | Transcription factor E2F2 | Q14209 | Homo sapiens |
| 209 | ACP3 | Prostatic acid phosphatase | P15309 | Homo sapiens |
| 210 | CTSD | Cathepsin D | P07339 | Homo sapiens |
| 211 | IGFBP3 | Insulin-like growth factor-binding protein 3 | P17936 | Homo sapiens |
| 212 | IGF2 | Insulin-like growth factor II | P01344 | Homo sapiens |
| 213 | CD40LG | CD40 ligand | P29965 | Homo sapiens |
| 214 | IRF1 | Interferon regulatory factor 1 | P10914 | Homo sapiens |
| 215 | ERBB3 | Receptor tyrosine-protein kinase erbB-3 | P21860 | Homo sapiens |
| 216 | PCOLCE | Procollagen C-endopeptidase enhancer 1 | Q15113 | Homo sapiens |
| 217 | NPEPPS | Puromycin-sensitive aminopeptidase | P55786 | Homo sapiens |
| 218 | HK2 | Hexokinase-2 | P52789 | Homo sapiens |
| 219 | NKX3-1 | Homeobox protein Nkx-3.1 | Q99801 | Homo sapiens |
| 220 | RASA1 | Ras GTPase-activating protein 1 | P20936 | Homo sapiens |
| 221 | NR3C1 | Glucocorticoid receptor | P04150 | Homo sapiens |
| 222 | ADH1C | Alcohol dehydrogenase 1C | P00326 | Homo sapiens |
| 223 | IGHG1 | Ig gamma-1 chain C region | P01857 | Homo sapiens |
| 224 | ADRA2A | Alpha-2A adrenergic receptor | P08913 | Homo sapiens |
| 225 | LTA4H | Leukotriene A-4 hydrolase | P09960 | Homo sapiens |
| 226 | MAOA | Amine oxidase [flavin-containing] A | P21397 | Homo sapiens |
| 227 | CTRB1 | Chymotrypsinogen B | P17538 | Homo sapiens |
| 228 | ADRB1 | Beta-1 adrenergic receptor | P08588 | Homo sapiens |
| 229 | SLC6A1 | GABA transporter 1 | P30531 | Homo sapiens |
| 230 | GABBR2 | GABA-B receptor | O75899 | Homo sapiens |
| 231 | GABRR1 | GABA receptor rho-1 subunit | P24046 | Homo sapiens |
| 232 | SLC6A11 | GABA transporter 3 | P48066 | Homo sapiens |
| 233 | SLC6A13 | GABA transporter 2 | Q9NSD5 | Homo sapiens |
| 234 | SLC6A12 | Betaine transporter | P48065 | Homo sapiens |
| 235 | GRIK1 | Glutamate receptor ionotropic kainate 1 | P39086 | Homo sapiens |
| 236 | GRIA1 | Glutamate receptor ionotropic, AMPA 1 | P42261 | Homo sapiens |
| 237 | GRIK5 | Glutamate receptor ionotropic kainate 5 | Q16478 | Homo sapiens |
| 238 | SLC1A1 | Excitatory amino acid transporter 3 | P43005 | Homo sapiens |
| 239 | GRM4 | Metabotropic glutamate receptor 4 | Q14833 | Homo sapiens |
| 240 | GRIA4 | Glutamate receptor ionotropic, AMPA 4 | P48058 | Homo sapiens |
| 241 | GRM5 | Metabotropic glutamate receptor 5 | P41594 | Homo sapiens |
| 242 | GRM8 | Metabotropic glutamate receptor 8 | O00222 | Homo sapiens |
| 243 | GRIK2 | Glutamate receptor ionotropic kainate 2 | Q13002 | Homo sapiens |
| 244 | GRIK3 | Glutamate receptor ionotropic kainate 3 | Q13003 | Homo sapiens |
| 245 | GRM1 | Metabotropic glutamate receptor 1 | Q13255 | Homo sapiens |
| 246 | GRM7 | Metabotropic glutamate receptor 7 | Q14831 | Homo sapiens |
| 247 | HDAC3 | Histone deacetylase 3 | O15379 | Homo sapiens |
| 248 | BBOX1 | Gamma-butyrobetaine dioxygenase | O75936 | Homo sapiens |
| 249 | CACNA2D1 | Voltage-gated calcium channel alpha2 | P54289 | Homo sapiens |
| 250 | SLC7A5 | L-type amino acid transporter 1 | Q01650 | Homo sapiens |
| 251 | ADORA3 | Adenosine A3 receptor | P0DMS8 | Homo sapiens |
| 252 | KDM4E | Lysine-specific demethylase 4D-like | B2RXH2 | Homo sapiens |
| 253 | FYN | Tyrosine-protein kinase FYN | P06241 | Homo sapiens |
| 254 | TACR1 | Neurokinin 1 receptor | P25103 | Homo sapiens |
| 255 | TH | Tyrosine 3-hydroxylase | P07101 | Homo sapiens |
| 256 | KDM4C | Lysine-specific demethylase 4C | Q9H3R0 | Homo sapiens |
| 257 | LCK | Tyrosine-protein kinase LCK | P06239 | Homo sapiens |
| 258 | ADRA2B | Alpha-2b adrenergic receptor | P18089 | Homo sapiens |
| 259 | CPB2 | Carboxypeptidase B2 isoform A | Q96IY4 | Homo sapiens |
| 260 | ANPEP | Aminopeptidase N | P15144 | Homo sapiens |
| 261 | ENPEP | Aminopeptidase A | Q07075 | Homo sapiens |
| 262 | KDM1A | Lysine-specific histone demethylase 1 | O60341 | Homo sapiens |
| 263 | CPA3 | Mast cell carboxypeptidase A | P15088 | Homo sapiens |
| 264 | TAAR1 | Trace amine-associated receptor 1 | Q96RJ0 | Homo sapiens |
| 265 | SLC15A1 | Oligopeptide transporter small intestine isoform | P46059 | Homo sapiens |
| 266 | CPB1 | Carboxypeptidase B | P15086 | Homo sapiens |
| 267 | PEPD | Xaa-Pro dipeptidase | P12955 | Homo sapiens |
| 268 | RNPEP | Aminopeptidase B | Q9H4A4 | Homo sapiens |
| 269 | OAT | Ornithine aminotransferase, mitochondrial | P04181 | Homo sapiens |
| 270 | KMO | Kynurenine 3-monooxygenase | O15229 | Homo sapiens |
| 271 | KYNU | Kynureninase | Q16719 | Homo sapiens |
| 272 | BHMT2 | S-methylmethionine--homocysteine S-methyltransferase BHMT2 | Q9H2M3 | Homo sapiens |
| 273 | GRM2 | Metabotropic glutamate receptor 2 | Q14416 | Homo sapiens |
| 274 | GRM3 | Metabotropic glutamate receptor 3 | Q14832 | Homo sapiens |
| 275 | GRM6 | Metabotropic glutamate receptor 6 | O15303 | Homo sapiens |
| 276 | SLC1A2 | Excitatory amino acid transporter 2 | P43004 | Homo sapiens |
| 277 | SLC22A6 | Solute carrier family 22 member 6 | Q4U2R8 | Homo sapiens |
| 278 | PLG | Plasminogen | P00747 | Homo sapiens |
| 279 | SHBG | Testis-specific androgen-binding protein | P04278 | Homo sapiens |
| 280 | THRA | Thyroid hormone receptor alpha | P10827 | Homo sapiens |
| 281 | THRB | Thyroid hormone receptor beta-1 | P10828 | Homo sapiens |
| 282 | PTPRA | Receptor-type tyrosine-protein phosphatase alpha | P18433 | Homo sapiens |
| 283 | NR1H4 | Bile acid receptor FXR | Q96RI1 | Homo sapiens |
| 284 | APEX1 | DNA-(apurinic or apyrimidinic site) lyase | P27695 | Homo sapiens |
| 285 | MAPKAPK2 | MAP kinase-activated protein kinase 2 | P49137 | Homo sapiens |
| 286 | GRB2 | Growth factor receptor-bound protein 2 | P62993 | Homo sapiens |
| 287 | KIF11 | Kinesin-like protein 1 | P52732 | Homo sapiens |
| 288 | EGLN1 | Egl nine homolog 1 | Q9GZT9 | Homo sapiens |
| 289 | ADORA2A | Adenosine A2a receptor | P29274 | Homo sapiens |
| 290 | ADORA2B | Adenosine A2b receptor | P29275 | Homo sapiens |
| 291 | PNP | Purine nucleoside phosphorylase | P00491 | Homo sapiens |
| 292 | PDK1 | Pyruvate dehydrogenase kinase isoform 1 | Q15118 | Homo sapiens |
| 293 | PIK3CA | PI3-kinase p110-alpha subunit | P42336 | Homo sapiens |
| 294 | PIM2 | Serine/threonine-protein kinase PIM2 | Q9P1W9 | Homo sapiens |
| 295 | PLK1 | Serine/threonine-protein kinase PLK1 | P53350 | Homo sapiens |
| 296 | BMP1 | Bone morphogenetic protein 1 | P13497 | Homo sapiens |
| 297 | AKR1B10 | Aldo-keto reductase family 1 member B10 | O60218 | Homo sapiens |
| 298 | HDAC6 | Histone deacetylase 6 | Q9UBN7 | Homo sapiens |
| 299 | HDAC1 | Histone deacetylase 1 | Q13547 | Homo sapiens |
| 300 | HSP90AA1 | Heat shock protein HSP 90-alpha | P07900 | Homo sapiens |
| 301 | SYK | Tyrosine-protein kinase SYK | P43405 | Homo sapiens |
| 302 | FCER2 | Immunoglobulin epsilon Fc receptor | P06734 | Homo sapiens |
| 303 | CDK5 | Cyclin-dependent kinase 5 | Q00535 | Homo sapiens |
| 304 | ALPL | Alkaline phosphatase, tissue-nonspecific isozyme | P05186 | Homo sapiens |
| 305 | ERN1 | Serine/threonine-protein kinase/endoribonuclease IRE1 | O75460 | Homo sapiens |
| 306 | RET | Kinesin-1 heavy chain/ Tyrosine-protein kinase receptor RET | P07949 | Homo sapiens |
| 307 | PDE4D | Phosphodiesterase 4D | Q08499 | Homo sapiens |
| 308 | CDK4 | Cyclin-dependent kinase 4 | P11802 | Homo sapiens |
| 309 | MTOR | Serine/threonine-protein kinase mTOR | P42345 | Homo sapiens |
| 310 | HDAC2 | Histone deacetylase 2 | Q92769 | Homo sapiens |
| 311 | AURKB | Serine/threonine-protein kinase Aurora-B | Q96GD4 | Homo sapiens |
| 312 | MYLK | Myosin light chain kinase, smooth muscle | Q15746 | Homo sapiens |
| 313 | GSK3A | Glycogen synthase kinase-3 alpha | P49840 | Homo sapiens |
| 314 | CA14 | Carbonic anhydrase XIV | Q9ULX7 | Homo sapiens |
| 315 | AURKA | Serine/threonine-protein kinase Aurora-A | O14965 | Homo sapiens |
| 316 | EPHB4 | Ephrin receptor | P54760 | Homo sapiens |
| 317 | MARK1 | Serine/threonine-protein kinase MARK1 | Q9P0L2 | Homo sapiens |
| 318 | AGPAT2 | 1-acylglycerol-3-phosphate O-acyltransferase beta | O15120 | Homo sapiens |
| 319 | BIRC2 | Baculoviral IAP repeat-containing protein 2 | Q13490 | Homo sapiens |
| 320 | PDE5A | Phosphodiesterase 5A | O76074 | Homo sapiens |
| 321 | GUSB | Beta-glucuronidase | P08236 | Homo sapiens |
| 322 | RPS6KB1 | Ribosomal protein S6 kinase 1 | P23443 | Homo sapiens |
| 323 | DUT | dUTP pyrophosphatase | P33316 | Homo sapiens |
| 324 | ECE1 | Endothelin-converting enzyme 1 | P42892 | Homo sapiens |
| 325 | HDAC10 | Histone deacetylase 10 | Q969S8 | Homo sapiens |
| 326 | PDE4B | Phosphodiesterase 4B | Q07343 | Homo sapiens |
| 327 | IMPDH1 | Inosine-5'-monophosphate dehydrogenase 1 | P20839 | Homo sapiens |
| 328 | IMPDH2 | Inosine-5'-monophosphate dehydrogenase 2 | P12268 | Homo sapiens |
| 329 | ABL1 | Tyrosine-protein kinase ABL | P00519 | Homo sapiens |
| 330 | CELA1 | Elastase 1 | Q9UNI1 | Homo sapiens |
| 331 | PREP | Prolyl endopeptidase | P48147 | Homo sapiens |
| 332 | CETP | Cholesteryl ester transfer protein | P11597 | Homo sapiens |
| 333 | STAT6 | Signal transducer and activator of transcription 6 | P42226 | Homo sapiens |
| 334 | WEE1 | Serine/threonine-protein kinase WEE1 | P30291 | Homo sapiens |
| 335 | PDE4A | Phosphodiesterase 4A | P27815 | Homo sapiens |
| 336 | PDE4C | Phosphodiesterase 4C | Q08493 | Homo sapiens |
| 337 | PTK2 | Focal adhesion kinase 1 | Q05397 | Homo sapiens |
| 338 | ROCK2 | Rho-associated protein kinase 2 | O75116 | Homo sapiens |
| 339 | SPHK2 | Sphingosine kinase 2 | Q9NRA0 | Homo sapiens |
| 340 | SPHK1 | Sphingosine kinase 1 | Q9NYA1 | Homo sapiens |
| 341 | DUSP3 | Dual specificity protein phosphatase 3 | P51452 | Homo sapiens |
| 342 | EP300 | Histone acetyltransferase p300 | Q09472 | Homo sapiens |
| 343 | TLR9 | Toll-like receptor (TLR7/TLR9) | Q9NR96 | Homo sapiens |
| 344 | PBK | PDZ-binding kinase | Q96KB5 | Homo sapiens |
| 345 | FLT3 | Tyrosine-protein kinase receptor FLT3 | P36888 | Homo sapiens |
| 346 | PIK3CD | PI3-kinase p110-delta subunit | O00329 | Homo sapiens |
| 347 | CFD | Complement factor D | P00746 | Homo sapiens |
| 348 | P2RX3 | P2X purinoceptor 3 | P56373 | Homo sapiens |
| 349 | PAK3 | Serine/threonine-protein kinase PAK 3 | O75914 | Homo sapiens |
| 350 | PIK3CB | PI3-kinase p110-beta subunit | P42338 | Homo sapiens |
| 351 | CDC25A | Dual specificity phosphatase Cdc25A | P30304 | Homo sapiens |
| 352 | PAK2 | Serine/threonine-protein kinase PAK 2 | Q13177 | Homo sapiens |
| 353 | PAK1 | Serine/threonine-protein kinase PAK 1 | Q13153 | Homo sapiens |
| 354 | BRAF | Serine/threonine-protein kinase B-raf | P15056 | Homo sapiens |
| 355 | RORC | Nuclear receptor ROR-gamma | P51449 | Homo sapiens |
| 356 | TYR | Tyrosinase | P14679 | Homo sapiens |
| 357 | CCR4 | C-C chemokine receptor type 4 | P51679 | Homo sapiens |
| 358 | LRRK2 | Leucine-rich repeat serine/threonine-protein kinase 2 | Q5S007 | Homo sapiens |
| 359 | GRK7 | G protein-coupled receptor kinase 7 | Q8WTQ7 | Homo sapiens |
| 360 | HIPK4 | Homeodomain-interacting protein kinase 4 | Q8NE63 | Homo sapiens |
| 361 | TAOK2 | Serine/threonine-protein kinase TAO2 | Q9UL54 | Homo sapiens |
| 362 | PIK3C2G | Phosphatidylinositol-4-phosphate 3-kinase C2 domain-containing subunit gamma | O75747 | Homo sapiens |
| 363 | PIP4K2C | Phosphatidylinositol-5-phosphate 4-kinase type-2 gamma | Q8TBX8 | Homo sapiens |
| 364 | CSF1R | Macrophage colony stimulating factor receptor | P07333 | Homo sapiens |
| 365 | FLT1 | Vascular endothelial growth factor receptor 1 | P17948 | Homo sapiens |
| 366 | CASK | Peripheral plasma membrane protein CASK | O14936 | Homo sapiens |
| 367 | PIP5K1C | Phosphatidylinositol-4-phosphate 5-kinase type-1 gamma | O60331 | Homo sapiens |
| 368 | DNM1 | Dynamin-1 | Q05193 | Homo sapiens |
| 369 | FNTA | Protein farnesyltransferase | P49354 | Homo sapiens |
| 370 | PDGFRB | Platelet-derived growth factor receptor beta | P09619 | Homo sapiens |
| 371 | MIF | Macrophage migration inhibitory factor | P14174 | Homo sapiens |
| 372 | BMP4 | Bone morphogenetic protein 4 | P12644 | Homo sapiens |
| 373 | ADAM10 | ADAM10 | O14672 | Homo sapiens |
| 374 | PLAA | Phospholipase A-2-activating protein | Q9Y263 | Homo sapiens |
| 375 | CAMK2D | CaM kinase II | Q13557 | Homo sapiens |
| 376 | TYMS | Thymidylate synthase | P04818 | Homo sapiens |
| 377 | MMP16 | Matrix metalloproteinase 16 | P51512 | Homo sapiens |
| 378 | PAK4 | Serine/threonine-protein kinase PAK 4 | O96013 | Homo sapiens |
| 379 | COMT | Catechol O-methyltransferase | P21964 | Homo sapiens |
| 380 | ALOX5AP | 5-lipoxygenase activating protein | P20292 | Homo sapiens |
| 381 | NOX4 | NADPH oxidase 4 | Q9NPH5 | Homo sapiens |
| 382 | HDAC11 | Histone deacetylase 11 | Q96DB2 | Homo sapiens |
| 383 | ALDH2 | Aldehyde dehydrogenase | P05091 | Homo sapiens |
| 384 | PTGER2 | Prostanoid EP2 receptor | P43116 | Homo sapiens |
| 385 | PIM3 | Serine/threonine-protein kinase PIM3 | Q86V86 | Homo sapiens |
| 386 | NCOR2 | Histone deacetylase 3/Nuclear receptor corepressor 2 (HDAC3/NCoR2) | Q9Y618 | Homo sapiens |
| 387 | JAK3 | Tyrosine-protein kinase JAK3 | P52333 | Homo sapiens |
| 388 | ALK | ALK tyrosine kinase receptor | Q9UM73 | Homo sapiens |
| 389 | TGFBR1 | TGF-beta receptor type I | P36897 | Homo sapiens |
| 390 | ACVR1 | Activin receptor type-1 | Q04771 | Homo sapiens |
| 391 | STAT3 | Signal transducer and activator of transcription 3 | P40763 | Homo sapiens |
| 392 | HSD17B3 | Estradiol 17-beta-dehydrogenase 3 | P37058 | Homo sapiens |
| 393 | HPGD | 15-hydroxyprostaglandin dehydrogenase [NAD+] | P15428 | Homo sapiens |
| 394 | NCOR1 | Histone deacetylase 3/NCoR1 | O75376 | Homo sapiens |
| 395 | HPGDS | Hematopoietic prostaglandin D synthase | O60760 | Homo sapiens |
| 396 | CCNE1 | Cyclin-dependent kinase 2/cyclin E1 | P24864 | Homo sapiens |
| 397 | FAAH | Anandamide amidohydrolase | O00519 | Homo sapiens |
| 398 | SAE1 | SUMO-activating enzyme | Q9UBE0 | Homo sapiens |
| 399 | ROCK1 | Rho-associated protein kinase 1 | Q13464 | Homo sapiens |
| 400 | LYN | Tyrosine-protein kinase Lyn | P07948 | Homo sapiens |
| 401 | TXK | Tyrosine-protein kinase TXK | P42681 | Homo sapiens |
| 402 | PSMB8 | Proteasome subunit beta type-8 | P28062 | Homo sapiens |
| 403 | PFKFB3 | 6-phosphofructo-2-kinase | Q16875 | Homo sapiens |
| 404 | TGM2 | Protein-glutamine gamma-glutamyltransferase | P21980 | Homo sapiens |
| 405 | TRPM8 | Transient receptor potential cation channel subfamily M member 8 | Q7Z2W7 | Homo sapiens |
| 406 | AGTR2 | Angiotensin II receptor | P50052 | Homo sapiens |
| 407 | MMP8 | Matrix metalloproteinase 8 | P22894 | Homo sapiens |
| 408 | HDAC8 | Histone deacetylase 8 | Q9BY41 | Homo sapiens |
| 409 | MCL1 | Induced myeloid leukemia cell differentiation protein Mcl-1 | Q07820 | Homo sapiens |
| 410 | MMP7 | Matrix metalloproteinase 7 | P09237 | Homo sapiens |
| 411 | KDR | Vascular endothelial growth factor receptor 2 | P35968 | Homo sapiens |
| 412 | PDE7A | Phosphodiesterase 7A | Q13946 | Homo sapiens |
| 413 | CTSK | Cathepsin K | P43235 | Homo sapiens |
| 414 | FKBP1A | FK506-binding protein 1A | P62942 | Homo sapiens |
| 415 | CAPN1 | Calpain 1 | P07384 | Homo sapiens |
| 416 | NPY1R | Neuropeptide Y receptor type 1 | P25929 | Homo sapiens |
| 417 | HTR1A | Serotonin 1a (5-HT1a) receptor | P08908 | Homo sapiens |
| 418 | HSD11B1 | 11-beta-hydroxysteroid dehydrogenase 1 | P28845 | Homo sapiens |
| 419 | PTGES | Prostaglandin E synthase | O14684 | Homo sapiens |
| 420 | MAP2K1 | Dual specificity mitogen-activated protein kinase kinase 1 | Q02750 | Homo sapiens |
| 421 | LYPLA1 | Acyl-protein thioesterase 1 | O75608 | Homo sapiens |
| 422 | LYPLA2 | Acyl-protein thioesterase 2 | O95372 | Homo sapiens |
| 423 | POLA1 | DNA polymerase alpha subunit | P09884 | Homo sapiens |
| 424 | HSD17B1 | Estradiol 17-beta-dehydrogenase 1 | P14061 | Homo sapiens |
| 425 | CA2 | Carbonic anhydrase II | P00918 | Homo sapiens |
| 426 | CA6 | Carbonic anhydrase VI | P23280 | Homo sapiens |
| 427 | CA4 | Carbonic anhydrase IV | P22748 | Homo sapiens |
| 428 | WNT3A | Protein Wnt-3a | P56704 | Homo sapiens |
| 429 | TNKS2 | Tankyrase-2 | Q9H2K2 | Homo sapiens |
| 430 | CNR1 | Cannabinoid receptor 1 | P21554 | Homo sapiens |
| 431 | CNR2 | Cannabinoid receptor 2 | P34972 | Homo sapiens |
| 432 | PRKDC | DNA-dependent protein kinase | P78527 | Homo sapiens |
| 433 | ITK | Tyrosine-protein kinase ITK/TSK | Q08881 | Homo sapiens |
| 434 | IGF1R | Insulin-like growth factor I receptor | P08069 | Homo sapiens |
| 435 | ADAMTS5 | ADAMTS5 | Q9UNA0 | Homo sapiens |
| 436 | ADAMTS4 | ADAMTS4 | O75173 | Homo sapiens |
| 437 | ST6GAL1 | Beta-galactoside alpha-2,6-sialyltransferase 1 | P15907 | Homo sapiens |
| 438 | KIF5B | Kinesin-1 heavy chain | P33176 | Homo sapiens |
| 439 | JAK1 | Tyrosine-protein kinase JAK1 | P23458 | Homo sapiens |
| 440 | KCNJ1 | ATP-sensitive inward rectifier potassium channel 1 | P48048 | Homo sapiens |
| 441 | ADA | Adenosine deaminase | P00813 | Homo sapiens |
| 442 | PER2 | Period circadian protein homolog 2 | O15055 | Homo sapiens |
| 443 | CA1 | Carbonic anhydrase I | P00915 | Homo sapiens |
| 444 | BRD4 | Bromodomain-containing protein 4 | O60885 | Homo sapiens |
| 445 | BRD2 | Bromodomain-containing protein 2 | P25440 | Homo sapiens |
| 446 | BRD3 | Bromodomain-containing protein 3 | Q15059 | Homo sapiens |
| 447 | CYP51A1 | Cytochrome P450 51 | Q16850 | Homo sapiens |
| 448 | CCR1 | C-C chemokine receptor type 1 | P32246 | Homo sapiens |
| 449 | LIMK1 | LIM domain kinase 1 | P53667 | Homo sapiens |
| 450 | CRHR1 | Corticotropin releasing factor receptor 1 | P34998 | Homo sapiens |
| 451 | RPS6KA3 | Ribosomal protein S6 kinase alpha 3 | P51812 | Homo sapiens |
| 452 | MDM2 | p53-binding protein Mdm-2 | Q00987 | Homo sapiens |
| 453 | SORD | Sorbitol dehydrogenase | Q00796 | Homo sapiens |
| 454 | JAK2 | Tyrosine-protein kinase JAK2 | O60674 | Homo sapiens |
| 455 | PRKCZ | Protein kinase C (PKC) | Q05513 | Homo sapiens |
| 456 | TYK2 | Tyrosine-protein kinase TYK2 | P29597 | Homo sapiens |
| 457 | TYRO3 | Tyrosine-protein kinase receptor TYRO3 | Q06418 | Homo sapiens |
| 458 | PTGFR | Prostanoid FP receptor | P43088 | Homo sapiens |
| 459 | NTRK1 | Nerve growth factor receptor Trk-A | P04629 | Homo sapiens |
| 460 | MKNK2 | MAP kinase signal-integrating kinase 2 | Q9HBH9 | Homo sapiens |
| 461 | TAB1 | TGF-beta-activated kinase 1 | Q15750 | Homo sapiens |
| 462 | MAP3K7 | Mitogen-activated protein kinase kinase kinase 7 | O43318 | Homo sapiens |
| 463 | TTR | Transthyretin | P02766 | Homo sapiens |
| 464 | DYRK1A | Dual-specificity tyrosine-phosphorylation regulated kinase 1A | Q13627 | Homo sapiens |
| 465 | PDF | Peptide deformylase mitochondrial | Q9HBH1 | Homo sapiens |
| 466 | ALOX12 | Arachidonate 12-lipoxygenase | P18054 | Homo sapiens |
| 467 | KCNK3 | Potassium channel subfamily K member 3 | O14649 | Homo sapiens |
| 468 | YES1 | Tyrosine-protein kinase YES | P07947 | Homo sapiens |
| 469 | SRC | Tyrosine-protein kinase SRC | P12931 | Homo sapiens |
| 470 | FAP | Fibroblast activation protein alpha | Q12884 | Homo sapiens |
| 471 | PRCP | Lysosomal Pro-X carboxypeptidase | P42785 | Homo sapiens |
| 472 | PDE2A | Phosphodiesterase 2A | O00408 | Homo sapiens |
| 473 | VHL | Von Hippel-Lindau disease tumor suppressor | P40337 | Homo sapiens |
| 474 | ATR | Serine-protein kinase ATR | Q13535 | Homo sapiens |
| 475 | NAMPT | Nicotinamide phosphoribosyltransferase | P43490 | Homo sapiens |
| 476 | MAPK10 | c-Jun N-terminal kinase 3 | P53779 | Homo sapiens |
| 477 | CDK8 | CDK8 | P49336 | Homo sapiens |
| 478 | SLC33A1 | Acetyl-coenzyme A transporter 1 | O00400 | Homo sapiens |
| 479 | CALCRL | Calcitonin gene-related peptide type 1 receptor | Q16602 | Homo sapiens |
| 480 | IL6ST | Interleukin-6 receptor subunit beta | P40189 | Homo sapiens |
| 481 | PIK3C2B | Phosphatidylinositol-4-phosphate 3-kinase C2 domain-containing beta polypeptide | O00750 | Homo sapiens |
| 482 | ERCC5 | DNA excision repair protein ERCC-5 | P28715 | Homo sapiens |
| 483 | FEN1 | Flap endonuclease 1 | P39748 | Homo sapiens |
| 484 | ADORA1 | Adenosine A1 receptor | P30542 | Homo sapiens |
| 485 | CA12 | Carbonic anhydrase XII | O43570 | Homo sapiens |
| 486 | CA9 | Carbonic anhydrase IX | Q16790 | Homo sapiens |
| 487 | TNKS | Tankyrase-1 | O95271 | Homo sapiens |
| 488 | ABCG2 | ATP-binding cassette sub-family G member 2 | Q9UNQ0 | Homo sapiens |
| 489 | TBXAS1 | Thromboxane-A synthase | P24557 | Homo sapiens |
| 490 | TACR2 | Neurokinin 2 receptor | P21452 | Homo sapiens |
| 491 | PLA2G2A | Phospholipase A2 group IIA | P14555 | Homo sapiens |
| 492 | CALM1 | Calmodulin | P0DP23 | Homo sapiens |
| 493 | CBR1 | Carbonyl reductase [NADPH] 1 | P16152 | Homo sapiens |
| 494 | ABCB1 | P-glycoprotein 1 | P08183 | Homo sapiens |
| 495 | CA7 | Carbonic anhydrase VII | P43166 | Homo sapiens |
| 496 | CDK6 | Cyclin-dependent kinase 6 | Q00534 | Homo sapiens |
| 497 | CFTR | Cystic fibrosis transmembrane conductance regulator | P13569 | Homo sapiens |
| 498 | APP | Beta amyloid A4 protein | P05067 | Homo sapiens |
| 499 | NTRK2 | Neurotrophic tyrosine kinase receptor type 2 | Q16620 | Homo sapiens |
| 500 | PTPRS | Receptor-type tyrosine-protein phosphatase S | Q13332 | Homo sapiens |
| 501 | AMY1A | AMY1C | P04745 | Homo sapiens |
| 502 | PARP2 | Poly [ADP-ribose] polymerase 2 | Q9UGN5 | Homo sapiens |
| 503 | GLO1 | Glyoxalase I | Q04760 | Homo sapiens |
| 504 | MMP12 | Matrix metalloproteinase 12 | P39900 | Homo sapiens |
| 505 | ARG1 | Arginase-1 | P05089 | Homo sapiens |
| 506 | ALOX15 | Arachidonate 15-lipoxygenase | P16050 | Homo sapiens |
| 507 | BCHE | Butyrylcholinesterase | P06276 | Homo sapiens |
| 508 | CSNK2A1 | Casein kinase II alpha | P68400 | Homo sapiens |
| 509 | GRK6 | G protein-coupled receptor kinase 6 | P43250 | Homo sapiens |
| 510 | SIRT2 | NAD-dependent deacetylase sirtuin 2 | Q8IXJ6 | Homo sapiens |
| 511 | DRD2 | Dopamine D2 receptor | P14416 | Homo sapiens |
| 512 | NAE1 | NEDD8-activating enzyme E1 regulatory subunit | Q13564 | Homo sapiens |
| 513 | TNFRSF1A | Tumor necrosis factor receptor R1 | P19438 | Homo sapiens |
| 514 | TERT | Telomerase reverse transcriptase | O14746 | Homo sapiens |
| 515 | SIGMAR1 | Sigma opioid receptor | Q99720 | Homo sapiens |
| 516 | MGLL | Monoglyceride lipase | Q99685 | Homo sapiens |
| 517 | CYP17A1 | Cytochrome P450 17A1 | P05093 | Homo sapiens |
| 518 | CXCR2 | Interleukin-8 receptor B | P25025 | Homo sapiens |
| 519 | ALDH3A1 | Aldehyde dehydrogenase dimeric NADP-preferring | P30838 | Homo sapiens |
| 520 | MTNR1A | Melatonin receptor 1A | P48039 | Homo sapiens |
| 521 | MTNR1B | Melatonin receptor 1B | P49286 | Homo sapiens |
| 522 | PIN1 | Peptidyl-prolyl cis-trans isomerase NIMA-interacting 1 | Q13526 | Homo sapiens |
| 523 | PLK3 | Serine/threonine-protein kinase PLK3 | Q9H4B4 | Homo sapiens |
| 524 | DHODH | Dihydroorotate dehydrogenase | Q02127 | Homo sapiens |
| 525 | TRPV3 | Transient receptor potential cation channel subfamily V member 3 | Q8NET8 | Homo sapiens |
| 526 | OPRK1 | Kappa Opioid receptor | P41145 | Homo sapiens |
| 527 | PTAFR | Platelet activating factor receptor | P25105 | Homo sapiens |
| 528 | HSD17B2 | Estradiol 17-beta-dehydrogenase 2 | P37059 | Homo sapiens |
| 529 | SCN9A | Sodium channel protein type IX alpha subunit | Q15858 | Homo sapiens |
| 530 | HCRTR2 | Orexin receptor 2 | O43614 | Homo sapiens |
| 531 | HCRTR1 | Orexin receptor 1 | O43613 | Homo sapiens |
| 532 | KCNA5 | Voltage-gated potassium channel subunit Kv1.5 | P22460 | Homo sapiens |
| 533 | CREBBP | CREB-binding protein/p53 | Q92793 | Homo sapiens |
| 534 | EPHX1 | Epoxide hydrolase 1 | P07099 | Homo sapiens |
| 535 | UTS2R | Urotensin II receptor | Q9UKP6 | Homo sapiens |
| 536 | CES2 | Carboxylesterase 2 | O00748 | Homo sapiens |
| 537 | P2RX7 | P2X purinoceptor 7 | Q99572 | Homo sapiens |
| 538 | RPS6KA5 | Ribosomal protein S6 kinase alpha 5 | O75582 | Homo sapiens |
| 539 | TNNC1 | Troponin, cardiac muscle | P63316 | Homo sapiens |
| 540 | FGFR1 | Fibroblast growth factor receptor 1 | P11362 | Homo sapiens |
| 541 | HRH3 | Histamine H3 receptor | Q9Y5N1 | Homo sapiens |
| 542 | TTK | Dual specificity protein kinase TTK | P33981 | Homo sapiens |
| 543 | IDO1 | Indoleamine 2,3-dioxygenase | P14902 | Homo sapiens |
| 544 | ELANE | Leukocyte elastase | P08246 | Homo sapiens |
| 545 | PTK2B | Protein tyrosine kinase 2 beta | Q14289 | Homo sapiens |
| 546 | MAPK14 | MAP kinase p38 alpha | Q16539 | Homo sapiens |
| 547 | TRPV1 | Vanilloid receptor | Q8NER1 | Homo sapiens |
| 548 | ACPP | Prostatic acid phosphatase | P15309 | Homo sapiens |
| 549 | EIF2AK1 | Eukaryotic translation initiation factor 2-alpha kinase 1 | Q9BQI3 | Homo sapiens |
| 550 | CYP11B1 | Cytochrome P450 11B1 | P15538 | Homo sapiens |
| 551 | CYP11B2 | Cytochrome P450 11B2 | P19099 | Homo sapiens |
| 552 | HRH4 | Histamine H4 receptor | Q9H3N8 | Homo sapiens |
| 553 | TSPO | Translocator protein | P30536 | Homo sapiens |
| 554 | PRKCG | Protein kinase C gamma | P05129 | Homo sapiens |
| 555 | DCTPP1 | dCTP pyrophosphatase 1 | Q9H773 | Homo sapiens |
| 556 | PTPN1 | Protein-tyrosine phosphatase 1B | P18031 | Homo sapiens |
| 557 | TUBB1 | Tubulin beta-1 chain | Q9H4B7 | Homo sapiens |
| 558 | DAO | D-amino-acid oxidase | P14920 | Homo sapiens |
| 559 | ALDH5A1 | Succinate semialdehyde dehydrogenase | P51649 | Homo sapiens |
| 560 | EEF2K | Serine/threonine-protein kinase EEF2K | O00418 | Homo sapiens |
| 561 | DHFR | Dihydrofolate reductase | P00374 | Homo sapiens |
| 562 | FADS1 | Fatty acid desaturase 1 | O60427 | Homo sapiens |
| 563 | CISD1 | CDGSH iron-sulfur domain-containing protein 1 | Q9NZ45 | Homo sapiens |
| 564 | LTB4R | Leukotriene B4 receptor 1 | Q15722 | Homo sapiens |
| 565 | CYP27A1 | Sterol 26-hydroxylase, mitochondrial | Q02318 | Homo sapiens |
| 566 | PLEC | Plectin | Q15149 | Homo sapiens |
| 567 | CSNK1A1 | Casein kinase I alpha | P48729 | Homo sapiens |
| 568 | CSNK1D | Casein kinase I delta | P48730 | Homo sapiens |
| 569 | ALPG | Alkaline phosphatase placental-like | P10696 | Homo sapiens |
| 570 | LNPEP | Cystinyl aminopeptidase | Q9UIQ6 | Homo sapiens |
| 571 | QPCT | Glutaminyl-peptide cyclotransferase | Q16769 | Homo sapiens |
| 572 | HTR2B | Serotonin 2b (5-HT2b) receptor | P41595 | Homo sapiens |
| 573 | STS | Steryl-sulfatase | P08842 | Homo sapiens |
| 574 | CTSV | Cathepsin (V and K) | O60911 | Homo sapiens |
| 575 | NAT1 | Arylamine N-acetyltransferase 1 | P18440 | Homo sapiens |
| 576 | GCGR | Glucagon receptor | P47871 | Homo sapiens |
| 577 | CDC25B | Dual specificity phosphatase Cdc25B | P30305 | Homo sapiens |
| 578 | GPBAR1 | G-protein coupled bile acid receptor 1 | Q8TDU6 | Homo sapiens |
| 579 | SHH | Sonic hedgehog protein | Q15465 | Homo sapiens |
| 580 | UGT2B7 | UDP-glucuronosyltransferase 2B7 | P16662 | Homo sapiens |
| 581 | NPC1L1 | Niemann-Pick C1-like protein 1 | Q9UHC9 | Homo sapiens |
| 582 | NR1H3 | LXR-alpha | Q13133 | Homo sapiens |
| 583 | G6PD | Glucose-6-phosphate 1-dehydrogenase | P11413 | Homo sapiens |
| 584 | GABBR1 | GABA-B receptor | Q9UBS5 | Homo sapiens |
| 585 | POLB | DNA polymerase beta | P06746 | Homo sapiens |
| 586 | EPHX2 | Epoxide hydratase | P34913 | Homo sapiens |
| 587 | SERPINA6 | Corticosteroid binding globulin | P08185 | Homo sapiens |
| 588 | LIPE | Hormone sensitive lipase | Q05469 | Homo sapiens |
| 589 | CD38 | Lymphocyte differentiation antigen CD38 | P28907 | Homo sapiens |
| 590 | C5AR1 | C5a anaphylatoxin chemotactic receptor | P21730 | Homo sapiens |
| 591 | HCK | Tyrosine-protein kinase HCK | P08631 | Homo sapiens |
| 592 | FGR | Tyrosine-protein kinase FGR | P09769 | Homo sapiens |
| 593 | EPHA3 | Ephrin type-A receptor 3 | P29320 | Homo sapiens |
| 594 | DDR2 | Discoidin domain-containing receptor 2 | Q16832 | Homo sapiens |
| 595 | BTK | Tyrosine-protein kinase BTK | Q06187 | Homo sapiens |
| 596 | DDR1 | Epithelial discoidin domain-containing receptor 1 | Q08345 | Homo sapiens |
| 597 | GPR88 | Probable G-protein coupled receptor 88 | Q9GZN0 | Homo sapiens |
| 598 | EZH2 | EZH2 | Q15910 | Homo sapiens |
| 599 | PDGFRA | Platelet-derived growth factor receptor | P16234 | Homo sapiens |
| 600 | CX3CR1 | C-X3-C chemokine receptor 1 | P49238 | Homo sapiens |
| 601 | EPHB3 | Ephrin type-B receptor 3 | P54753 | Homo sapiens |
| 602 | NPY2R | Neuropeptide Y receptor type 2 | P49146 | Homo sapiens |
| 603 | MPEG1 | Macrophage-expressed gene 1 protein | Q2M385 | Homo sapiens |
| 604 | MMP13 | Matrix metalloproteinase 13 | P45452 | Homo sapiens |
| 605 | PCNA | Proliferating cell nuclear antigen | P12004 | Homo sapiens |
| 606 | PDE3B | Phosphodiesterase 3B | Q13370 | Homo sapiens |
| 607 | ADAM17 | ADAM17 | P78536 | Homo sapiens |
| 608 | PRF1 | Perforin-1 | P14222 | Homo sapiens |
| 609 | VCP | Transitional endoplasmic reticulum ATPase | P55072 | Homo sapiens |
| 610 | MMP14 | Matrix metalloproteinase 14 | P50281 | Homo sapiens |
| 611 | KDM4A | Lysine-specific demethylase 4A | O75164 | Homo sapiens |
| 612 | PBRM1 | Protein polybromo-1 | Q86U86 | Homo sapiens |
| 613 | SMARCA4 | Transcription activator BRG1 | P51532 | Homo sapiens |
| 614 | CLK1 | Dual specificty protein kinase CLK1 | P49759 | Homo sapiens |
| 615 | DYRK1B | Dual specificity tyrosine-phosphorylation-regulated kinase 1B | Q9Y463 | Homo sapiens |
| 616 | HDAC5 | Histone deacetylase 5 | Q9UQL6 | Homo sapiens |
| 617 | NOX1 | NADPH oxidase 1 | Q9Y5S8 | Homo sapiens |
| 618 | MME | Neprilysin | P08473 | Homo sapiens |
| 619 | CYP2C19 | Cytochrome P450 2C19 | P33261 | Homo sapiens |
| 620 | SLC1A3 | Excitatory amino acid transporter 1 | P43003 | Homo sapiens |
| 621 | PI4KB | PI4-kinase beta subunit | Q9UBF8 | Homo sapiens |
| 622 | CLK4 | Dual specificity protein kinase CLK4 | Q9HAZ1 | Homo sapiens |
| 623 | CLK2 | Dual specificity protein kinase CLK2 | P49760 | Homo sapiens |
| 624 | CLK3 | Dual specificity protein kinase CLK3 | P49761 | Homo sapiens |
| 625 | SMARCA2 | Probable global transcription activator SNF2L2 | P51531 | Homo sapiens |
| 626 | LPL | Lipoprotein lipase | P06858 | Homo sapiens |
| 627 | LIPC | Hepatic lipase | P11150 | Homo sapiens |
| 628 | PLA2G7 | LDL-associated phospholipase A2 | Q13093 | Homo sapiens |
| 629 | SLC27A4 | Fatty acid transport protein 4 | Q6P1M0 | Homo sapiens |
| 630 | SREBF2 | Sterol regulatory element-binding protein 2 | Q12772 | Homo sapiens |
| 631 | GLI2 | Zinc finger protein GLI2 | P10070 | Homo sapiens |
| 632 | GLI1 | Zinc finger protein GLI1 | P08151 | Homo sapiens |
| 633 | CCR2 | C-C chemokine receptor type 2 | P41597 | Homo sapiens |
| 634 | PTPN2 | T-cell protein-tyrosine phosphatase | P17706 | Homo sapiens |
| 635 | SRD5A1 | Steroid 5-alpha-reductase 1 | P18405 | Homo sapiens |
| 636 | SRD5A2 | Steroid 5-alpha-reductase 2 | P31213 | Homo sapiens |
| 637 | CA13 | Carbonic anhydrase XIII | Q8N1Q1 | Homo sapiens |
| 638 | CA5B | Carbonic anhydrase VB | Q9Y2D0 | Homo sapiens |
| 639 | CA5A | Carbonic anhydrase VA | P35218 | Homo sapiens |
