# Supplementary material for: Network Pharmacology Identifies the Mechanisms of Action of Tongxie Anchang Decoction in the Treatment of Irritable Bowel Syndrome with Diarrhea Predominant
Source: Evid Based Complement Alternat Med. 2020 Nov 17;2020:2723705. doi: 10.1155/2020/2723705 (PMC7685835; doi:10.1155/2020/2723705)
Supplement: Supplementary Materials — 1: the active compounds of TXACD. Supplementary materials 2: the information on potential targets of TXACD. Supplementary materials 3: the information on potential targets of IBS-D. Supplementary materials 4: raw data of GO enrichment analysis. Supplementary materials 5: raw data of KEGG enrichment analysis. [file 2723705.f1.zip › supplementary material 3.docx]

Supplementary material 3 Information on potential targets of IBS-D

| Gene Symbol | Relevance score |
| --- | --- |
| TNF | 91.64 |
| IL6 | 85.39 |
| IL10 | 81.44 |
| ALB | 67.49 |
| IL1B | 66.98 |
| TP53 | 65.26 |
| NOD2 | 65.16 |
| CD40LG | 64.78 |
| IFNG | 64.38 |
| TLR4 | 63.47 |
| INS | 55.39 |
| FOXP3 | 53.98 |
| CCR6 | 53.69 |
| SLC6A4 | 52.06 |
| MPO | 48.63 |
| TLR2 | 48.62 |
| EGFR | 48.61 |
| FLNA | 48.08 |
| PTEN | 47.31 |
| KRAS | 45.28 |
| AKT1 | 45.07 |
| CTLA4 | 44.66 |
| HLA-DRB1 | 44.32 |
| ABCB1 | 44.19 |
| BDNF | 44.11 |
| CXCL8 | 43.7 |
| TGFB1 | 43.69 |
| MSH2 | 42.92 |
| IL13 | 42.91 |
| SMAD4 | 42.59 |
| MMP9 | 42.55 |
| IL1RN | 42.42 |
| MLH1 | 42.25 |
| STAT3 | 42.2 |
| CCL2 | 41.85 |
| EPCAM | 41.67 |
| ICAM1 | 41.52 |
| IL2RA | 41.09 |
| HRAS | 40.48 |
| FBN1 | 40.21 |
| NF1 | 39.81 |
| ATM | 39.69 |
| COMT | 39.57 |
| TNFRSF1A | 39.07 |
| EGF | 38.38 |
| PIK3CA | 38.1 |
| MSH6 | 37.79 |
| TH | 37.4 |
| HLA-B | 37.31 |
| SCN5A | 37.19 |
| ACE | 36.87 |
| CFTR | 36.85 |
| APC | 36.82 |
| CRP | 36.8 |
| ELANE | 36.78 |
| LEP | 36.73 |
| IGF1 | 36.36 |
| ERBB2 | 36.18 |
| IL2 | 35.99 |
| ERCC6 | 35.96 |
| FAS | 35.94 |
| OCLN | 35.09 |
| CDKN2A | 34.71 |
| BRAF | 34.47 |
| STAT1 | 34.46 |
| BRCA2 | 34.42 |
| UGT1A1 | 34.21 |
| SST | 34.2 |
| IL4 | 34.2 |
| CD79A | 34.17 |
| CD40 | 34.12 |
| HLA-DQB1 | 33.98 |
| TNFRSF1B | 33.96 |
| MMP2 | 33.91 |
| NOS2 | 33.87 |
| WT1 | 33.79 |
| KIT | 33.76 |
| PTPN11 | 33.71 |
| CCND1 | 33.61 |
| FASLG | 33.56 |
| FOS | 33.56 |
| BRCA1 | 33.54 |
| STK11 | 33.15 |
| IL23R | 33.13 |
| HTR3A | 33.13 |
| HTR2A | 32.88 |
| NOTCH1 | 32.88 |
| MTOR | 32.83 |
| AIRE | 32.68 |
| CD46 | 32.58 |
| TGFBR2 | 32.37 |
| MECP2 | 32.31 |
| CDH1 | 32.09 |
| APOE | 32.04 |
| TLR5 | 31.98 |
| GDNF | 31.96 |
| CACNA1A | 31.89 |
| PMS2 | 31.81 |
| FMR1 | 31.59 |
| PPARG | 31.24 |
| FGFR1 | 31.18 |
| RET | 31.06 |
| POLG | 31.06 |
| DOCK8 | 31.05 |
| TTR | 31.02 |
| SLC26A3 | 30.74 |
| MIR21 | 30.65 |
| HLA-A | 30.63 |
| SLC9A3 | 30.38 |
| TLR3 | 30.18 |
| BMPR1A | 29.89 |
| MMP1 | 29.73 |
| B2M | 29.72 |
| ATRX | 29.69 |
| PRTN3 | 29.66 |
| ELN | 29.61 |
| PTH | 29.56 |
| JAK2 | 29.49 |
| CD28 | 29.48 |
| IL17A | 29.46 |
| SOD1 | 29.42 |
| CCL11 | 29.4 |
| MAPK1 | 29.37 |
| CXCL12 | 29.25 |
| GUCY2C | 29.21 |
| MBL2 | 29.2 |
| MIR155 | 29.16 |
| TGFB2 | 29.13 |
| CXCR4 | 29.09 |
| AR | 29.07 |
| MEN1 | 29.07 |
| ADA | 28.95 |
| VDR | 28.82 |
| POMC | 28.8 |
| NR3C1 | 28.79 |
| RAF1 | 28.71 |
| TGFBR1 | 28.63 |
| CCL3 | 28.53 |
| PTPN22 | 28.5 |
| SLC2A1 | 28.5 |
| H19 | 28.38 |
| MME | 28.25 |
| NLRP3 | 28.23 |
| SNCA | 27.97 |
| DRD2 | 27.86 |
| TERT | 27.82 |
| WAS | 27.77 |
| GNAS | 27.76 |
| MEFV | 27.7 |
| PDGFRB | 27.7 |
| KITLG | 27.69 |
| SKIV2L | 27.64 |
| NRAS | 27.61 |
| CCK | 27.59 |
| MAPT | 27.59 |
| MIR17 | 27.54 |
| CREBBP | 27.44 |
| SMAD3 | 27.38 |
| EDN1 | 27.19 |
| PTPRC | 27.05 |
| SPP1 | 26.96 |
| MT-CYB | 26.94 |
| VEGFA | 26.92 |
| HLA-DQA1 | 26.87 |
| SRC | 26.87 |
| LTA | 26.84 |
| DNMT1 | 26.82 |
| RETN | 26.79 |
| BMP4 | 26.79 |
| VWF | 26.74 |
| CSF3 | 26.65 |
| CHEK2 | 26.6 |
| IL10RA | 26.57 |
| LCK | 26.51 |
| CASP8 | 26.42 |
| CTNNB1 | 26.34 |
| CD4 | 26.31 |
| PTCH1 | 26.23 |
| IL5 | 26.21 |
| IL7R | 26.2 |
| EZH2 | 26.15 |
| RAD51 | 26.11 |
| LRBA | 26.09 |
| STXBP2 | 26.07 |
| DNMT3B | 26.04 |
| ATP7A | 26 |
| C3 | 25.99 |
| BTK | 25.91 |
| CDKN1A | 25.86 |
| SMARCA4 | 25.85 |
| IL2RB | 25.85 |
| KCNQ1 | 25.83 |
| F5 | 25.83 |
| IGF2 | 25.67 |
| WRN | 25.57 |
| MUTYH | 25.53 |
| FGFR2 | 25.52 |
| HLA-C | 25.51 |
| LIG4 | 25.48 |
| PRF1 | 25.41 |
| COL5A1 | 25.36 |
| IL18 | 25.26 |
| GPT | 25.17 |
| DES | 25.11 |
| HSPG2 | 25.1 |
| TFRC | 25.09 |
| VIP | 25.01 |
| H2AC18 | 24.97 |
| GHRL | 24.74 |
| IL7 | 24.72 |
| CDC42 | 24.7 |
| GFAP | 24.68 |
| TAC1 | 24.67 |
| TGFB3 | 24.53 |
| U2AF1 | 24.51 |
| PDGFRA | 24.45 |
| SERPINA1 | 24.37 |
| IL1A | 24.36 |
| CHGA | 24.29 |
| ITGAM | 24.27 |
| PYY | 24.12 |
| HMOX1 | 24.09 |
| MEG3 | 24.08 |
| SPINT2 | 24.07 |
| SH2D1A | 24.06 |
| DBH | 24.05 |
| DMD | 24.01 |
| TSC2 | 24 |
| IRF1 | 23.87 |
| JAG1 | 23.82 |
| SPINK5 | 23.81 |
| HFE | 23.8 |
| FN1 | 23.73 |
| EDNRB | 23.72 |
| SHH | 23.71 |
| F2 | 23.67 |
| PRNP | 23.66 |
| TBX1 | 23.66 |
| TYMP | 23.63 |
| XIAP | 23.61 |
| REN | 23.58 |
| MIR34A | 23.55 |
| CD55 | 23.53 |
| MIR20A | 23.47 |
| SLC5A1 | 23.43 |
| WFS1 | 23.42 |
| IFNA1 | 23.37 |
| MIR200A | 23.31 |
| CHAT | 23.29 |
| DNMT3A | 23.23 |
| MIR146A | 23.21 |
| MYD88 | 23.18 |
| CD8A | 23.1 |
| NTRK1 | 23.09 |
| GLI3 | 23.05 |
| MDM2 | 22.93 |
| KMT2D | 22.89 |
| GH1 | 22.89 |
| DICER1 | 22.88 |
| CRYAA | 22.87 |
| ZAP70 | 22.84 |
| FGFR3 | 22.78 |
| CDK4 | 22.73 |
| IL4R | 22.71 |
| APP | 22.64 |
| MIR125A | 22.6 |
| CRH | 22.58 |
| ADAM17 | 22.57 |
| SLC25A13 | 22.49 |
| EP300 | 22.48 |
| SYP | 22.48 |
| JAK3 | 22.43 |
| HPRT1 | 22.35 |
| LOX | 22.22 |
| IGF2R | 22.12 |
| CSF2 | 22.1 |
| GJA1 | 22.05 |
| SI | 22.01 |
| MIR126 | 22.01 |
| MLN | 21.98 |
| HELLS | 21.94 |
| SDHD | 21.92 |
| NGF | 21.89 |
| MIR223 | 21.82 |
| LCT | 21.82 |
| CD19 | 21.81 |
| KCNH2 | 21.77 |
| TMPRSS15 | 21.71 |
| CASR | 21.7 |
| WNT5A | 21.69 |
| MVK | 21.62 |
| ALK | 21.6 |
| CYP3A4 | 21.54 |
| MAP2K1 | 21.53 |
| TRPA1 | 21.52 |
| ICOSLG | 21.48 |
| HTT | 21.48 |
| CXCL10 | 21.47 |
| CALCA | 21.43 |
| SLC10A2 | 21.42 |
| CDKN2B | 21.41 |
| PIK3R1 | 21.41 |
| SCN4A | 21.29 |
| MT-CO1 | 21.29 |
| PTGS2 | 21.25 |
| MPZ | 21.24 |
| SLC6A2 | 21.22 |
| COL5A2 | 21.18 |
| THBD | 21.15 |
| TLR9 | 21.13 |
| CBL | 21.06 |
| MIR145 | 21.04 |
| CIITA | 21.03 |
| EDN3 | 21.02 |
| GREM1 | 21.02 |
| NOS3 | 20.98 |
| KCNJ11 | 20.93 |
| OPRM1 | 20.88 |
| NFKB1 | 20.88 |
| C4A | 20.88 |
| AKT3 | 20.81 |
| PAX3 | 20.81 |
| MIR221 | 20.81 |
| PIK3C2A | 20.77 |
| MIR18A | 20.72 |
| LYST | 20.68 |
| HP | 20.67 |
| ENG | 20.66 |
| TPH1 | 20.61 |
| MTHFR | 20.58 |
| CCL5 | 20.57 |
| GAST | 20.47 |
| NOTCH2 | 20.47 |
| NEUROG3 | 20.43 |
| PRKN | 20.37 |
| MIR132 | 20.27 |
| ADIPOQ | 20.22 |
| SIL1 | 20.2 |
| IL12B | 20.15 |
| MYLK | 20.11 |
| ESR1 | 20.05 |
| RYR2 | 20.05 |
| AURKA | 20.04 |
| TGM2 | 20.01 |
| PIK3R2 | 19.94 |
| MIR140 | 19.94 |
| NBN | 19.88 |
| TP63 | 19.87 |
| UBE3A | 19.86 |
| MGMT | 19.86 |
| MT-ATP6 | 19.79 |
| PDCD1 | 19.73 |
| PLA2G2A | 19.69 |
| FGF2 | 19.69 |
| LRRK2 | 19.68 |
| TCF4 | 19.67 |
| SDHB | 19.65 |
| NPY | 19.6 |
| G6PD | 19.59 |
| LRP5 | 19.56 |
| GNRH1 | 19.56 |
| PSEN1 | 19.55 |
| SCT | 19.54 |
| MITF | 19.5 |
| IRF5 | 19.43 |
| GATA4 | 19.41 |
| ATXN2 | 19.36 |
| ITGB2 | 19.34 |
| IL1R1 | 19.32 |
| IDH1 | 19.3 |
| PRRT2 | 19.28 |
| CNR1 | 19.26 |
| PSMB9 | 19.22 |
| HGF | 19.22 |
| AGTR1 | 19.18 |
| HTR1A | 19.15 |
| GATA1 | 19.11 |
| TF | 19.06 |
| CDKN1B | 19.06 |
| ERBB3 | 19.02 |
| HLA-DPB1 | 18.98 |
| PROM1 | 18.96 |
| XBP1 | 18.93 |
| CCND2 | 18.9 |
| EPO | 18.89 |
| IL21 | 18.88 |
| CYP21A2 | 18.87 |
| MIR214 | 18.85 |
| PMS1 | 18.83 |
| NRXN1 | 18.81 |
| HTR4 | 18.79 |
| ARX | 18.74 |
| L1CAM | 18.72 |
| SCN10A | 18.71 |
| CASP3 | 18.7 |
| TACR1 | 18.69 |
| ACTA1 | 18.68 |
| GJB1 | 18.59 |
| ERCC2 | 18.57 |
| APOA1 | 18.45 |
| CNTNAP2 | 18.45 |
| MIR127 | 18.44 |
| SCN2A | 18.43 |
| FLT1 | 18.39 |
| SOX2 | 18.37 |
| STAT4 | 18.25 |
| IFIH1 | 18.23 |
| OTC | 18.23 |
| IL15 | 18.22 |
| STX11 | 18.18 |
| VCP | 18.16 |
| ABCC2 | 18.15 |
| BMP2 | 18.15 |
| KMT2A | 18.15 |
| IL3 | 18.1 |
| ADRB2 | 18.06 |
| DNAH8 | 18.05 |
| COL1A2 | 18.03 |
| SETBP1 | 18.02 |
| PMP22 | 17.99 |
| C9orf72 | 17.97 |
| TWIST1 | 17.96 |
| MIR222 | 17.89 |
| SELE | 17.86 |
| GCG | 17.84 |
| S100A9 | 17.84 |
| PRKAR1A | 17.78 |
| SLC17A5 | 17.75 |
| MAOA | 17.71 |
| TRPV1 | 17.71 |
| ALOX5 | 17.7 |
| VCAM1 | 17.7 |
| MYC | 17.68 |
| FHIT | 17.66 |
| CD36 | 17.63 |
| CDKL5 | 17.6 |
| MAP2K2 | 17.59 |
| RB1 | 17.58 |
| GJB2 | 17.58 |
| DGAT1 | 17.57 |
| CACNA1C | 17.54 |
| SOX9 | 17.54 |
| CYP19A1 | 17.52 |
| OGG1 | 17.51 |
| TSC1 | 17.5 |
| IGF1R | 17.5 |
| F3 | 17.45 |
| TIMP1 | 17.37 |
| ACADS | 17.34 |
| LTF | 17.3 |
| TTC7A | 17.3 |
| TOP1 | 17.25 |
| MRE11 | 17.23 |
| ACHE | 17.22 |
| MMP3 | 17.19 |
| IL17F | 17.19 |
| GATA3 | 17.19 |
| HLA-G | 17.14 |
| NR1H4 | 17.12 |
| SMARCB1 | 17.12 |
| STAT5B | 17.11 |
| CACNA1S | 17.08 |
| CFH | 17.06 |
| PRL | 17.05 |
| MIR29C | 17.03 |
| ERAP1 | 17.03 |
| CYP27B1 | 17.02 |
| NOTCH3 | 17 |
| BGLAP | 16.99 |
| HSPB1 | 16.99 |
| FLT3 | 16.98 |
| CRHR1 | 16.97 |
| PLAU | 16.92 |
| KMT2C | 16.92 |
| MET | 16.9 |
| APOB | 16.9 |
| PALB2 | 16.88 |
| IL12RB1 | 16.86 |
| SDHA | 16.85 |
| PHOX2B | 16.81 |
| HNF4A | 16.76 |
| AXIN2 | 16.75 |
| SELP | 16.75 |
| CYP17A1 | 16.73 |
| ACVRL1 | 16.72 |
| SQSTM1 | 16.72 |
| ITK | 16.71 |
| TNXB | 16.68 |
| TBX21 | 16.68 |
| PC | 16.68 |
| DDX58 | 16.51 |
| SCN9A | 16.5 |
| CX3CR1 | 16.5 |
| SERPINA3 | 16.48 |
| HLA-DPA1 | 16.47 |
| CCN2 | 16.43 |
| IFNA2 | 16.4 |
| CLCN2 | 16.4 |
| MED12 | 16.4 |
| COL2A1 | 16.39 |
| MIR483 | 16.38 |
| SERPINC1 | 16.36 |
| DSP | 16.34 |
| MRAP | 16.31 |
| MIR19A | 16.22 |
| IL1RAPL2 | 16.21 |
| CDSN | 16.19 |
| TNFAIP3 | 16.08 |
| GLI1 | 16.08 |
| DRD4 | 16.04 |
| FLCN | 16.03 |
| MUC1 | 15.95 |
| ERCC1 | 15.92 |
| MAPK14 | 15.91 |
| TYR | 15.89 |
| SAG | 15.86 |
| CAV1 | 15.82 |
| PAX6 | 15.82 |
| MIR24-1 | 15.77 |
| ESR2 | 15.71 |
| NTS | 15.69 |
| ABL1 | 15.67 |
| ABCB11 | 15.66 |
| NLRP12 | 15.6 |
| ITGB3 | 15.59 |
| TOP2A | 15.57 |
| FLNB | 15.55 |
| BAX | 15.53 |
| CREB1 | 15.53 |
| IGFBP3 | 15.48 |
| BCL2 | 15.44 |
| GZMB | 15.43 |
| FLG | 15.42 |
| AVP | 15.41 |
| ISL1 | 15.4 |
| SMARCA2 | 15.34 |
| CDH3 | 15.34 |
| ATXN7 | 15.32 |
| XK | 15.32 |
| S100B | 15.24 |
| CCR5 | 15.15 |
| CLCNKB | 15.14 |
| ATP8B1 | 15.12 |
| KDR | 15.08 |
| COX5A | 15.06 |
| CAT | 15.05 |
| PLG | 15.04 |
| CYP1B1 | 15.03 |
| TSHR | 15.03 |
| MIR200B | 15.02 |
| ODC1 | 14.96 |
| LPL | 14.93 |
| MIR150 | 14.92 |
| GSN | 14.9 |
| BRIP1 | 14.84 |
| RHO | 14.81 |
| KCNJ1 | 14.8 |
| SETD2 | 14.77 |
| CHEK1 | 14.72 |
| DPYD | 14.7 |
| KCNE1 | 14.64 |
| SERPINE1 | 14.61 |
| FTL | 14.6 |
| HIF1A | 14.6 |
| ETS1 | 14.56 |
| CD80 | 14.5 |
| CDKN3 | 14.46 |
| ACADM | 14.45 |
| KDM4C | 14.44 |
| SLC22A5 | 14.43 |
| BMP6 | 14.43 |
| APOH | 14.43 |
| GSTP1 | 14.34 |
| CASP1 | 14.33 |
| GUSB | 14.32 |
| MIR143 | 14.28 |
| RARB | 14.26 |
| MAPK8 | 14.24 |
| SELL | 14.24 |
| GSTM1 | 14.22 |
| DARS2 | 14.16 |
| UBE2L3 | 14.15 |
| RNASE3 | 14.13 |
| CXCL9 | 14.13 |
| GGT1 | 14.1 |
| FCGR2A | 14.07 |
| IL23A | 14.07 |
| PMM2 | 14.06 |
| MIR100 | 14.06 |
| ABCB4 | 14.02 |
| IRS1 | 14.02 |
| MIR96 | 14 |
| TYMS | 14 |
| ADAMTS13 | 13.99 |
| PCNA | 13.99 |
| KCNQ1OT1 | 13.98 |
| RAC1 | 13.98 |
| LOC110806262 | 13.96 |
| BTD | 13.96 |
| MIR29A | 13.94 |
| CPOX | 13.94 |
| COL17A1 | 13.9 |
| TKT | 13.88 |
| NTRK2 | 13.88 |
| SLC22A4 | 13.88 |
| IGHE | 13.87 |
| CYCS | 13.87 |
| HSPA4 | 13.87 |
| MIR10A | 13.82 |
| NT5E | 13.81 |
| AKT2 | 13.8 |
| MIR148A | 13.79 |
| POLE | 13.76 |
| PSMB8 | 13.76 |
| PPOX | 13.73 |
| MIR31 | 13.71 |
| MIR146B | 13.65 |
| COL11A1 | 13.61 |
| TNFSF13B | 13.61 |
| VIM | 13.61 |
| GPC3 | 13.59 |
| EDNRA | 13.59 |
| MYCN | 13.56 |
| NCAM1 | 13.56 |
| CYP1A2 | 13.56 |
| GRIN2B | 13.52 |
| CXCR3 | 13.49 |
| NLRP1 | 13.48 |
| ANXA5 | 13.44 |
| MIR9-1 | 13.43 |
| TRPV4 | 13.43 |
| MIR200C | 13.43 |
| NR5A1 | 13.42 |
| JUN | 13.41 |
| LEPR | 13.38 |
| ARID1A | 13.37 |
| TGFA | 13.37 |
| MIR203A | 13.37 |
| HBG2 | 13.37 |
| PRODH | 13.36 |
| STAR | 13.35 |
| MIF | 13.35 |
| EPAS1 | 13.33 |
| MIR22 | 13.31 |
| ITGAL | 13.31 |
| MIR378A | 13.3 |
| MAPK3 | 13.3 |
| NDUFV1 | 13.3 |
| S100A12 | 13.29 |
| KCNN3 | 13.26 |
| F8 | 13.25 |
| CCL4 | 13.21 |
| MIRLET7A1 | 13.21 |
| MMEL1 | 13.2 |
| KRT7 | 13.18 |
| NAGLU | 13.18 |
| CALR | 13.17 |
| VHL | 13.15 |
| MTR | 13.12 |
| TPH2 | 13.11 |
| CYP2D6 | 13.1 |
| MAF | 13.08 |
| GNB3 | 13.08 |
| PECAM1 | 13.06 |
| CHUK | 13.06 |
| ABCC8 | 13.05 |
| COMP | 13.04 |
| TYK2 | 13.04 |
| MIR142 | 13.02 |
| RAD21 | 12.98 |
| F9 | 12.98 |
| CRHR2 | 12.96 |
| GAPDH | 12.93 |
| AGT | 12.92 |
| NRTN | 12.92 |
| BARD1 | 12.92 |
| SLC12A3 | 12.88 |
| PNLIP | 12.85 |
| PPARA | 12.81 |
| DLL1 | 12.81 |
| ACTG2 | 12.8 |
| CD14 | 12.76 |
| ENO2 | 12.74 |
| MT-ATP8 | 12.74 |
| IFNB1 | 12.74 |
| IL9 | 12.73 |
| POLD1 | 12.72 |
| FLNC | 12.66 |
| DCTN1 | 12.66 |
| FLVCR1 | 12.6 |
| STX1A | 12.58 |
| MIR30B | 12.58 |
| AFP | 12.58 |
| GRB2 | 12.57 |
| ITGA4 | 12.57 |
| CDX2 | 12.56 |
| MIR199B | 12.56 |
| MMP14 | 12.55 |
| ATR | 12.52 |
| LIFR | 12.52 |
| MIR210 | 12.51 |
| MIR335 | 12.5 |
| TMPO | 12.49 |
| HSPD1 | 12.47 |
| SNAP25 | 12.47 |
| BAP1 | 12.45 |
| MIRLET7B | 12.42 |
| IL17RA | 12.4 |
| FLG2 | 12.4 |
| COQ2 | 12.36 |
| TOR1A | 12.35 |
| HSP90AA1 | 12.34 |
| BCL2L1 | 12.34 |
| CXCL1 | 12.33 |
| BUB1B | 12.33 |
| GRIN2A | 12.32 |
| ALDH18A1 | 12.3 |
| MIR23A | 12.29 |
| BCR | 12.26 |
| ADRA2A | 12.23 |
| CSF1 | 12.23 |
| ECE1 | 12.23 |
| CD274 | 12.23 |
| MTRR | 12.22 |
| AVPR2 | 12.22 |
| TCF7L2 | 12.22 |
| TPMT | 12.21 |
| LBR | 12.19 |
| IL11 | 12.19 |
| CHRM3 | 12.18 |
| PLA2G4A | 12.17 |
| HDAC9 | 12.17 |
| PEX6 | 12.13 |
| CTNND1 | 12.12 |
| HTR7 | 12.1 |
| FGF19 | 12.1 |
| VEGFC | 12.09 |
| PTGS1 | 12.08 |
| PRKD1 | 12.07 |
| THRB | 12.06 |
| SEMA3A | 12.06 |
| CA2 | 12.04 |
| MIR122 | 12.02 |
| IL22 | 12 |
| DSG2 | 12 |
| FLT4 | 11.99 |
| CP | 11.98 |
| HMGB1 | 11.97 |
| PTHLH | 11.94 |
| CTSG | 11.92 |
| ARSA | 11.92 |
| NFKBIA | 11.92 |
| MSH3 | 11.91 |
| MUC5AC | 11.88 |
| PSAP | 11.88 |
| PGR | 11.86 |
| TET2 | 11.85 |
| INSR | 11.84 |
| F2RL1 | 11.81 |
| GNRHR | 11.79 |
| KIR3DL1 | 11.79 |
| CCR3 | 11.75 |
| TLR1 | 11.74 |
| PRDM16 | 11.74 |
| MICA | 11.72 |
| YAP1 | 11.71 |
| JUP | 11.69 |
| ASCL1 | 11.67 |
| HLA-DRA | 11.66 |
| PRKCD | 11.66 |
| CCR1 | 11.62 |
| ST14 | 11.62 |
| DEFB4A | 11.62 |
| PLCB1 | 11.6 |
| IDO1 | 11.6 |
| CPT2 | 11.57 |
| THPO | 11.56 |
| SMAD2 | 11.56 |
| IL16 | 11.52 |
| TNFSF10 | 11.48 |
| KCNE3 | 11.47 |
| ABCG2 | 11.44 |
| KIF1B | 11.43 |
| LMOD1 | 11.41 |
| TNFRSF8 | 11.41 |
| GRP | 11.39 |
| GRN | 11.38 |
| CGA | 11.36 |
| HAMP | 11.36 |
| NFE2L2 | 11.35 |
| S100A8 | 11.3 |
| TCF3 | 11.3 |
| CASP9 | 11.28 |
| AHR | 11.28 |
| SCN1B | 11.27 |
| DUOX2 | 11.25 |
| TEK | 11.25 |
| CDK2 | 11.24 |
| SPG11 | 11.24 |
| AXIN1 | 11.23 |
| DDC | 11.2 |
| CD209 | 11.17 |
| SHBG | 11.14 |
| CD34 | 11.13 |
| PANK2 | 11.13 |
| PARK7 | 11.12 |
| PARP1 | 11.12 |
| STAT6 | 11.1 |
| CXCR2 | 11.09 |
| FGF7 | 11.09 |
| GFI1 | 11.09 |
| OPRK1 | 11.07 |
| MIR196A1 | 11.05 |
| MMP13 | 11.05 |
| EPRS1 | 10.99 |
| UCHL1 | 10.99 |
| H2AX | 10.98 |
| EZR | 10.97 |
| XRCC2 | 10.97 |
| IDUA | 10.94 |
| HTR3E | 10.93 |
| ALAD | 10.9 |
| CFB | 10.9 |
| IRF7 | 10.88 |
| SOCS1 | 10.88 |
| HNMT | 10.88 |
| ABCC1 | 10.88 |
| AQP4 | 10.87 |
| MIR30A | 10.86 |
| HDAC6 | 10.86 |
| FADD | 10.85 |
| CD86 | 10.79 |
| JAK1 | 10.73 |
| SLC12A1 | 10.73 |
| BIRC5 | 10.73 |
| ITPA | 10.71 |
| NR3C2 | 10.71 |
| KRT14 | 10.71 |
| FGFR4 | 10.7 |
| LRP6 | 10.69 |
| AIP | 10.68 |
| TNFRSF11B | 10.67 |
| DIABLO | 10.66 |
| ITGB4 | 10.62 |
| CFLAR | 10.61 |
| DDX3X | 10.61 |
| SOCS3 | 10.59 |
| DPP4 | 10.58 |
| TNFRSF10A | 10.58 |
| PLEC | 10.58 |
| ALX3 | 10.58 |
| IL6ST | 10.57 |
| SIRT1 | 10.57 |
| NOD1 | 10.56 |
| CLDN1 | 10.5 |
